# Differential co-expression networks of long non-coding RNAs and mRNAs in *Cleistogenes songorica* under water stress and during recovery

**DOI:** 10.1186/s12870-018-1626-5

**Published:** 2019-01-11

**Authors:** Qi Yan, Fan Wu, Zhuanzhuan Yan, Jie Li, Tiantian Ma, Yufei Zhang, Yufeng Zhao, Yanrong Wang, Jiyu Zhang

**Affiliations:** State Key Laboratory of Grassland Agro-ecosystems, Key Laboratory of Grassland Livestock Industry Innovation, Ministry of Agriculture and Rural Affairs, College of Pastoral Agriculture Science and Technology, Lanzhou University, Lanzhou, People’s Republic of China

**Keywords:** LncRNA, Water stress, *Cleistogenes songorica*, RNA-seq, Transcription factor, Conserved drought-responsive gene, Poaceae, Expression network

## Abstract

**Background:**

Water stress seriously constrains plant growth and yield. Long non-coding RNAs (lncRNAs) serve as versatile regulators in various biological regulatory processes. To date, the systematic screening and potential functions of lncRNA have not yet been characterized in *Cleistogenes songorica*, especially under water stress conditions.

**Results:**

In this study, we obtained the root and shoot transcriptomes of young *C. songorica* plants subjected to different degrees of water stress and recovery treatments by Illumina-based RNA-seq. A total of 3397 lncRNAs were identified through bioinformatics analysis. LncRNA differential expression analysis indicated that the higher response of roots compared to shoots during water stress and recovery. We further identified the 1644 transcription factors, 189 of which were corresponded to 163 lncRNAs in *C. songorica*. Though comparative analyses with major Poaceae species based on blast, 81 water stress-related orthologues regulated to lncRNAs were identified as a core of evolutionary conserved genes important to regulate water stress responses in the family. Among these target genes, two genes were found to be involved in the abscisic acid (ABA) signalling pathway, and four genes were enriched for starch and sucrose metabolism. Additionally, the 52 lncRNAs were predicted as target mimics for microRNAs (miRNAs) in *C. songorica*. RT-qPCR results suggested that MSTRG.43964.1 and MSTRG.4400.2 may regulate the expression of miRNA397 and miRNA166, respectively, as target mimics under water stress and during recovery. Finally, a co-expression network was constructed based on the lncRNAs, miRNAs, protein-coding genes (PCgenes) and transcription factors under water stress and during recovery in *C. songorica*.

**Conclusions:**

In *C. songorica*, lncRNAs, miRNAs, PCgenes and transcription factors constitute a complex transcriptional regulatory network which lncRNAs can regulate PCgenes and miRNAs under water stress and recovery. This study provides fundamental resources to deepen our knowledge on lncRNAs during ubiquitous water stress.

**Electronic supplementary material:**

The online version of this article (10.1186/s12870-018-1626-5) contains supplementary material, which is available to authorized users.

## Background

As the global population is growing, it will require a significant increase in agricultural production to meet global food needs for the next half century [1]. This challenge further exacerbates the intensity and frequency of extreme events [2, 3]. The global temperature is predicted to rise 3–6 °C by 2100, which may cause the duration and frequency of drought periods to increase [4, 5]. Currently, drought is an important abiotic stress for plants that constrains plant growth and yield around the world [6]. Unlike animals, plants cannot escape environmental pressures and are constantly exposed to various environments during their life cycles. To survive in these harsh environments, plants develop many resistance mechanisms. For example, the model plant *Arabidopsis thaliana* responds to abiotic stress by undergoing morphological and physiological characteristic changes [7]. Therefore, it is important to understand the molecular and physiological mechanisms that plants use to respond to drought stress.

A considerable portion of the eukaryotic genome can be transcribed into RNAs but will not be translated into proteins. Non-coding RNAs (ncRNAs) are a set of RNAs that cannot code for proteins. NcRNAs consist of housekeeping, regulatory and functionally unknown ncRNAs. Regulatory ncRNAs are usually grouped into groups, such as miRNAs, small interfering RNAs and lncRNAs, according to their lengths [8, 9]. In general, lncRNAs are longer than 200 nt in length and lack region of protein coding [10]. LncRNAs are generally classified into different types based on the relative location between PCgenes and lncRNAs in genome. For example, intronic lncRNAs and long intergenic non-coding RNAs (lincRNAs) are transcribed from intron and intergenic regions, respectively [11]. In *Arabidopsis*, more than 30% of lncRNAs are lincRNAs, though antisense lncRNAs are also abundant [12, 13]. Transcription analysis indicated that lncRNAs have low transcription level and specific expression patterns in plant tissue. Furthermore, lncRNAs also showed low conservation and be located to subcellular compartments. Compared to PCgenes, lncRNAs were initially considered to be inconsequential transcriptional “noise”.

However, increasing studies suggested that lncRNAs involved in multiple biological processes, such as flowering time, root organogenesis, photomorphogenesis and sexual reproduction [14–17]. Additionally, lncRNAs are recognized as playing key regulatory mechanism in plant under abiotic stresses [11]. For example, more than 1000 lncRNAs are regulated by salt stress and 318 lncRNAs respond to cold and/or drought stress in cotton and cassava, respectively [18, 19].

Functional analysis of some lncRNAs has indicated that they can regulate the expression of genes in close proximity (*cis-acting*) or at a distance (*trans-acting*) in the genome via numerous of mechanisms, including DNA methylation, histone modifications, and the activation/transportation of accessory proteins [13, 20, 21]. Compared to studies focused on lncRNAs in mammals, only a few studies have reported the functions of lncRNAs in plants, especially in grass [22, 23]. For example, *COLDAIR*, an intronic lncRNA, is transcribed from the first intron in *FLOWERING LOCUS C* (*FLC*) and has been identified to be associated with the silencing and epigenetic repression of *FLC* to regulate flowering time in *Arabidopsis* [14, 24]. *AtIPS1* and *At4* have been identified to act as target mimics of miR399 by binding to and sequestering miRNA399 and reducing the miRNA399-midiated cleavage of *PHO2*, which is important for phosphate uptake in *Arabidopsis* [25, 26]. In *Gossypium hirsutum*, lnc_883 may participate in regulating salt stress tolerance by modulating the expression of *Gh_D03G0339* [18]. Because lncRNAs have important functions in plants, several strategies have been employed to detect and discover novel lncRNAs [27]. Microarrays, tiling arrays and next generation sequencing have been used as high-throughput tools in genome-wide analyses to identify new transcripts. Thousands of lncRNA transcripts have been identified in several plant species. To date, more than 200,000 lncRNAs from 44 plant species have been found in the Green Non-Coding Database (GreeNC Database) [28].

*Cleistogenes songorica* is a C_4_ grass in the Poaceae family and an important perennial forage and ecological grass. *C. songorica* can grow in saline, semi-arid and desert areas in Northwest China, such as Inner Mongolia, where the average annual rainfall is 110 mm [29]. To study the drought tolerance mechanism of *C. songorica*, expression sequence tags (ESTs) from two organs were sequenced under drought stress [30]. Transforming the *C. songorica LEA* and *ALDH* genes into transgenic alfalfa and *Arabidopsis* improved resistance to drought and salt stress [31–34]. This evidence indicates that *C. songorica* is an ideal candidate plant system for the identification of drought tolerance-conferring genes. We have recently completed whole-genome sequencing of *C. songorica* (data not published). However, these studies did not explore and study lncRNAs and mRNAs under drought stress in *C. songorica*, and the mechanisms by which lncRNAs and PCgenes participate in drought tolerance remains obscure, demonstrating that it needs to be further explored. Here, we performed a genome-wide scanning study using strand-specific RNA-seq on 24 cDNA libraries to discover and characterize lncRNAs and mRNAs from *C. songorica* that are challenged by water stress and during recovery.

## Results

### Effects of water stress and recovery on photosynthesis

To examine the effects of water stress and during recovery on *C. songorica* photosynthesis processes, 8-week-old *C. songorica* seedings were subjected to different water stress and recovery treatments. Under water stress and during recovery conditions, the leaf relative water content (RWC), photosynthesis rate (Pn), intercellular CO2 concentration (Ci), stomatal conductance (Gs), transpiration rate (Tr) and chlorophyll content (Chl) showed a continuous decrease up to severe water stress, which were all significantly (*p* < 0.05) lower than the control groups (Additional file 1). Strikingly, *C. songorica* still maintained approximately 50% RWC under severe water stress (2% soil water content). Compared to the control groups, there were no significant differences in the RWC, Chl, Ci and Tr after 48 h of recovery. However, the Pn and Gs reached 80.43 and 62.2% of control group levels after 48 h of recovery treatment, respectively (Additional file 1). Finally, the control (CK), light water stress (LS), severe water stress (SS) and 48 h recovery (R) samples were used for high-throughput sequencing.

### Genome-wide identification and characterization of lncRNAs in *C. songorica*

In this study, we performed strand-specific RNA sequencing on 24 samples (four treatments, two tissues, three biological replicates). We obtained the clean reads after the removal of the low-quality reads from the RNA-seq data. To estimate the data quality, the Fast QC and GC contents were calculated from clean data. All clean datasets were mapped to the *C. songorica* genome using HISAT2 to reconstruct the *C. songorica* transcriptome. The mapping rates were mostly greater than 85%. The transcripts were assembled and annotated using StringTie. These results showed that the RNA-seq data were highly reliable (Additional file 2).

**Fig. 1:**
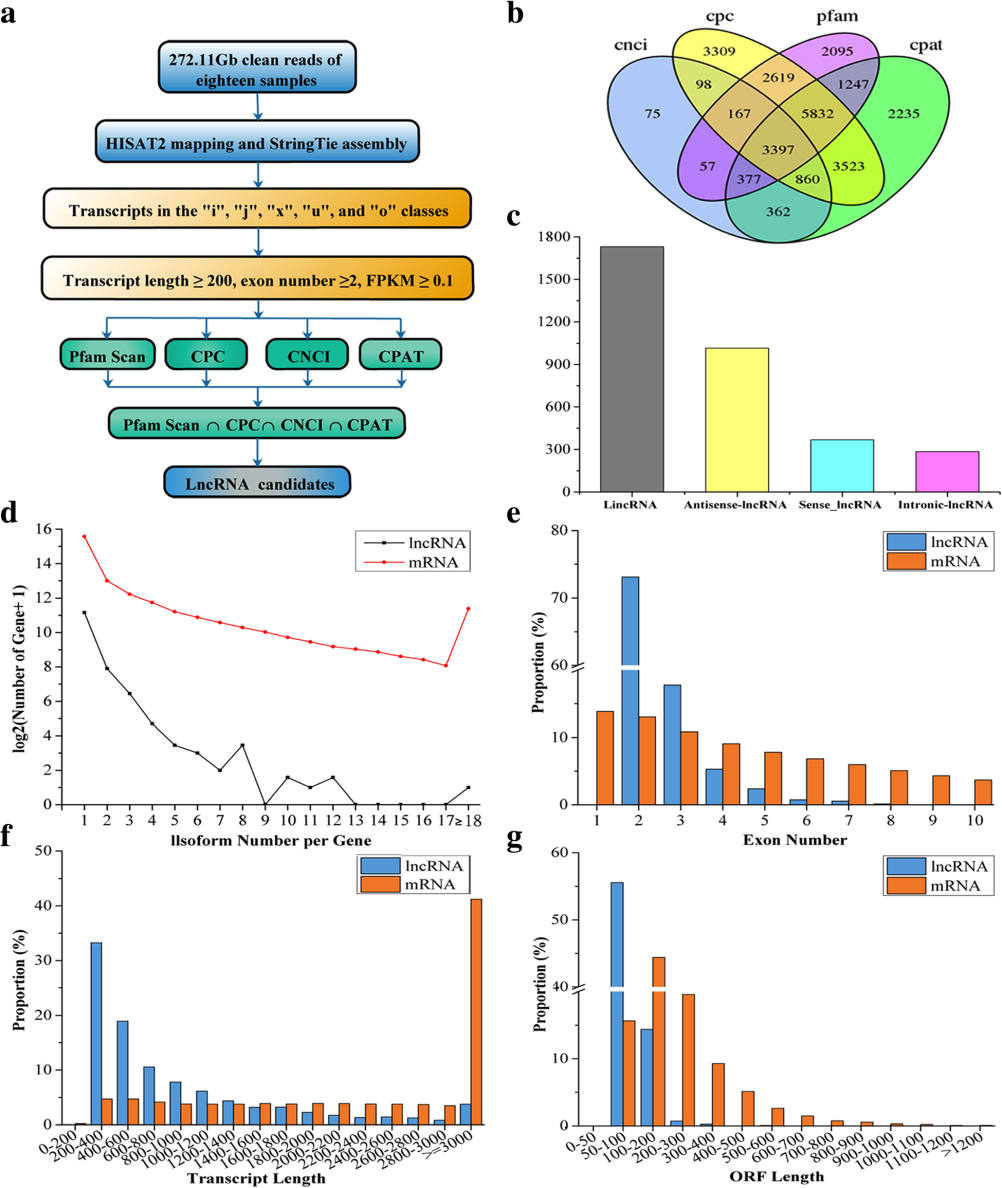
An integrative computational pipeline for the systematic identification and characteristics of lncRNAs in *C. songorica*. **a** Informatics pipeline for the identification of lncRNAs in *C. songorica*. **b** Venn chart showing the numbers of candidate lncRNAs filtered by the Pfam, CPC, CPAT and CNCI assemblies or by both assemblies. Pfam: Pfam Scan, CPC: Coding Potential Calculator, CPAT: Coding Potential Assessment Tool, CNCI: Coding-Non-Coding Index. **c** Composition of different types of lncRNAs. **d** Number distributions of spliced *C. songorica* lncRNAs and mRNAs. **e** The number of exons per transcription for mRNAs and lncRNAs. **f** Transcript size distributions for all mRNAs and lncRNAs. **g** Open reading frame (ORF) distributions for all mRNAs and lncRNAs

Identification of lncRNAs was executed according to the pipeline shown in Fig. 1a. Using this pipeline, 5397, 19,805, 15,791, and 17,833 candidate lncRNAs were predicted by with Coding-Non-Coding Index (CNCI), Coding Potential Calculator (CPC), Pfam Scan (Pfam) and Coding-Potential Assessment Tool (CPAT), respectively (Fig. 1b). LncRNAs can be divided into different categories based on their genomic location. In total, 3397 lncRNAs were obtained from the intersection of the four analysis methods, including 1730 lincRNAs, 1016 antisense lncRNAs, 284 intronic lncRNAs, and 367 sense lncRNAs (Fig. 1c).

We characterized the basic genomic features of the lncRNAs and mRNAs, including the transcript abundance, transcript length, exon number and ORF in *C. songorica* (Fig. 1d-g). We then estimated the expression level of each transcript using the fragments per kilobase of exon per million fragments mapped (FPKM) and found that lncRNAs were expressed at similar levels in the control and water stress groups. Interestingly, the overall expression levels of mRNAs were higher than lncRNAs (Additional file 3). Compared to the mRNAs from *C. songorica*, 78.5% of the lncRNAs were spliced in our study (Fig. 1d). Most of the lncRNAs (~ 75%) contained two exons, while mRNAs had more exons and exon numbers distributed in a wider range (Fig. 1e). The full and ORF length of *C. songorica* lncRNAs were shorter. For example, the ORF length of most lncRNAs (52.2%) was shorter than 600 nt, while only 21.3% of mRNA ORFs were shorter than 1200 nt (Fig. 1g). The majority of lncRNAs (52%) were 200 to 600 nt in lengths, while most mRNAs (41%) were longer than 3000 nt (Fig. 1f). As compared *C. songorica* lncRNAs with genomic sequences from two eudicot and two monocot species. The result showed that only a small portion of lncRNAs (from 0.2% between *C. songorica* and *Arabidopsis* to 3.8% between *C. songorica* and rice had significant hits, suggesting substantially low conservation (Additional file 4).

**Fig. 2:**
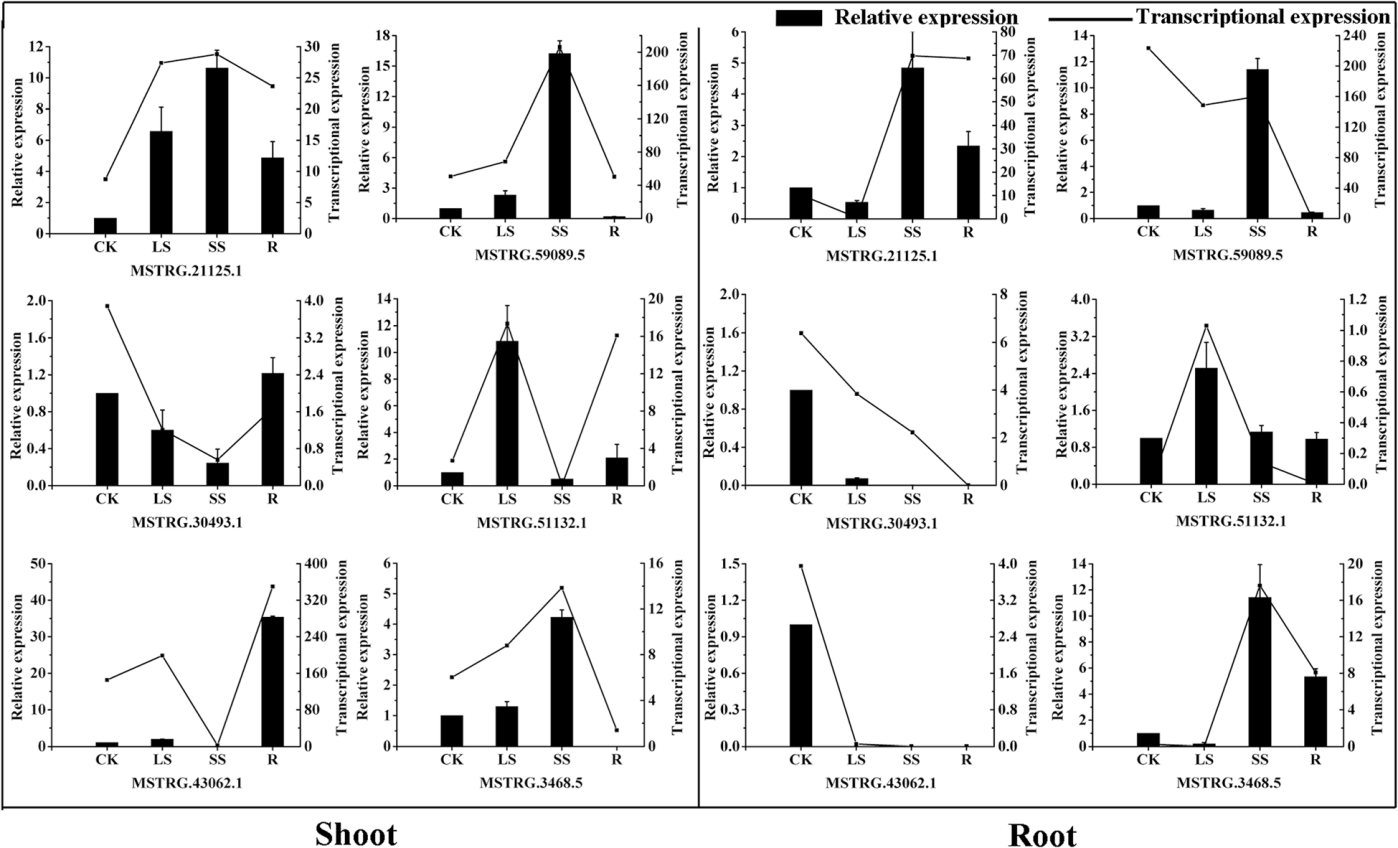
Confirmation of the expression patterns of lncRNAs using quantitative RT-PCR (CK: control, LS: light water stress, SS: severe water stress and R: 48 h recovery. The values shown are the means ± standard deviation of three replicates. *CsGAPDH* was used as the reference gene

To characterize the expression pattern of lncRNAs, 6 lncRNAs under water stress and recovery were randomly selected and analysed by RT-qPCR. As shown in Fig. 2, the expression patterns of the stress-responsive lncRNAs by RNA-seq and RT-qPCR were relatively consistent with similar trends, indicating that the lncRNAs expression based on RNA-seq data are reliable. Consequently, three and two lncRNAs were identified as up-regulated in shoot and root under water stress, respectively. Two lncRNAs were down-regulated in root under water stress and recovery (Fig. 2).

### Identification of differentially expressed genes (DEGs) and lncRNAs (DE-lncRNAs) by RNA-seq

**Fig. 3:**
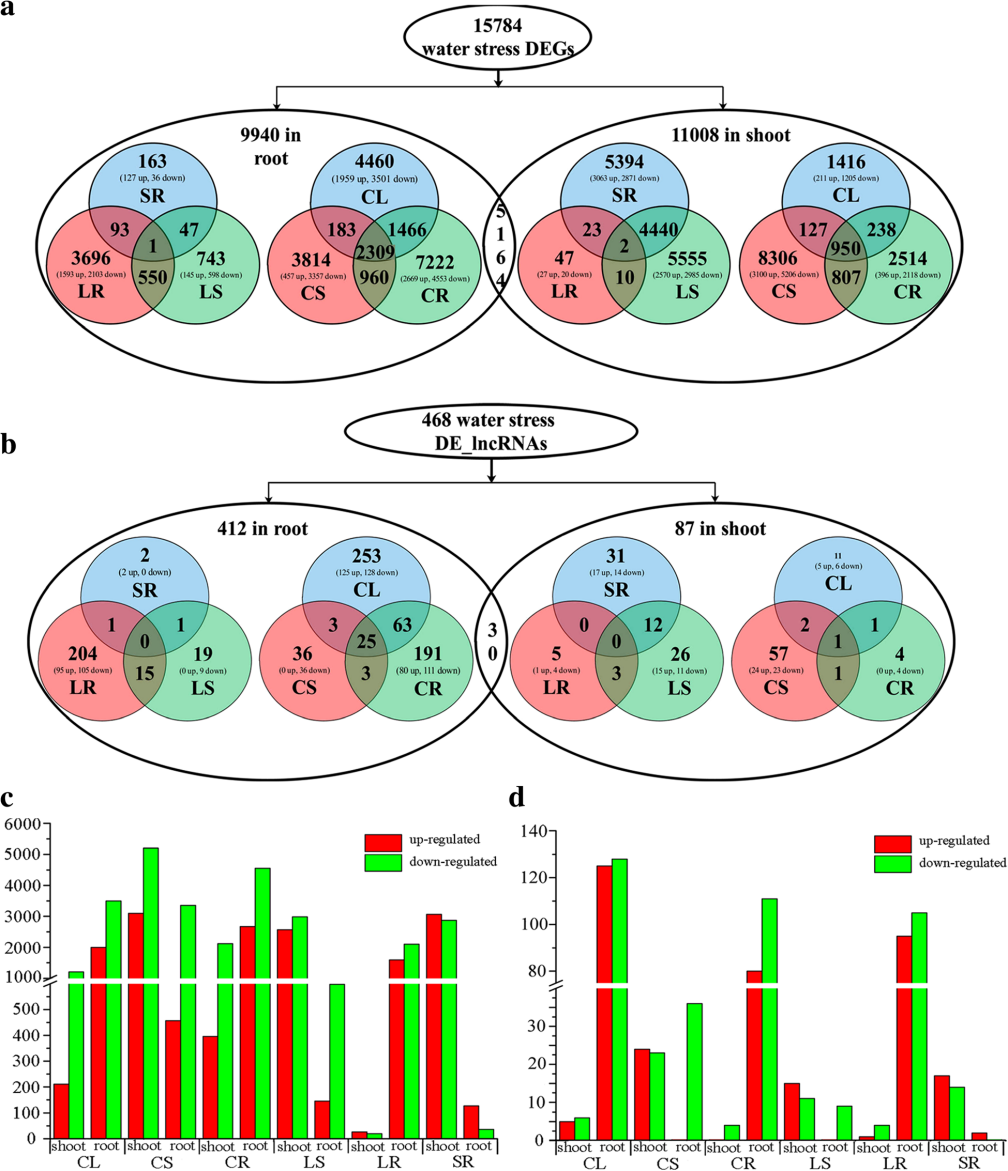
Summary of DEGs and DE-lncRNAs (Fold change ≥4; FDR ≤ 0.01) in roots and shoots of *C. songorica* upon water stress and recovery. **a** Number of genes upregulated / downregulated by water stress and recovery treatments under different conditions (*CL*: control vs. light water stress, *CS*: control vs. severe water stress, *CR*: control vs. recovery, *LS*: light water stress vs. severe water stress, *LR*: light water stress vs. recovery, *SR*: severe water stress vs. recovery) in root and shoot. **b** Number of lncRNAs upregulated / downregulated by water stress and recovery treatments under different conditions. **c** Number of regulated genes between different conditions. *Red bar* upregulated genes; *green bar* downregulated genes. **d** Number of regulated lncRNAs between different conditions. *Red bar* upregulated lncRNAs; *green bar* downregulated lncRNAs

Gene expression profiling of the water stress, recovery and control *C. songorica* plants at the four abovementioned physiological stages allowed us to identify PCgenes and lncRNAs whose expression levels were significantly changed upon water treatment. A total of 15,784 PCgenes and 468 lncRNAs showed differential expression for at least one of the three stress conditions (CK vs. LS, CK vs. SS, CK vs. R, LS vs. SS, LS vs. R, or SS vs. R), with roughly the same number of DEGs in shoots and roots (11,008 and 9940 DEGs, respectively; Fig. 3a). However, the number of DE-lncRNAs (87 and 412, respectively) were significantly different in the shoots and roots (Fig. 3b).

To investigate the relationship between the transcriptomes from different treatment samples, we performed a correlation analysis among on the normalized expression values from all the samples and generated heat maps (Additional file 5). The heat maps indicated that the CK, LS and R treatments were clustered together compared to the SS treatment in shoots (Additional file 5). However, the SS and R treatments clustered together compared to the LS and CK treatments in the roots (Additional file 5).

A detailed assessment of the number and identity of DEGs and DE-lncRNAs between the conditions for each organ confirmed this observation; in the shoots, there were 1416 DEGs and 11 DE-lncRNAs between the CK and LS conditions, 8306 DEGs and 57 DE-lncRNAs between the CK and SS conditions, 2514 DEGs and 4 DE-lncRNAs between the CK and R conditions, 5394 DEGs and 31 DE-lncRNAs between the SS and R conditions, and 47 DEGs and 5 DE-lnRNAs between the LS and R conditions, respectively. Similar trends were also observed for the DEGs and DE-lncRNAs in the roots (Additional files 6 and 7). By comparison, a total of 5164 common DEGs and 30 common DE-lncRNAs were identified between the organs (Fig. 3a and b). The overall direction in the expression variation was conserved between organs, with the majority of DEGs being downregulated rather than upregulated; the downregulated and upregulated DE-lncRNAs showed almost the same trends (Fig. 3c and d).

### Functional analysis of the DE-lncRNAs and DEGs

LncRNAs located upstream of genes may take part in transcriptional regulation by interaction with promoters or other *cis*-acting elements, and downstream lncRNAs may be involved in other regulatory activities. In this study, we annotated lncRNAs by scanning up to 100 kb upstream and downstream of genes, and analysing the complementary base-pairing between antisense lncRNAs and mRNAs using LncTar software [35]. In Table 1, 3332 upstream or downstream lncRNAs and 161 antisense lncRNAs interaction with 31,812 (60.7%) *cis*-target genes and 202 *trans*-target genes, respectively (Additional file 8). Furthermore, 27,455 (86.3%) *cis*-target genes and 166 (82.2%) *trans*-target genes were expressed under water stress and recovery. Among these, 9321 (29.3%) *cis*-target genes and 75 (37,1%) *trans*-target genes were differentially expressed under water stress and recovery (Table 1 and Additional file 8). To confirm the relationship of DE-lncRNA and target genes, three lncRNA and three target genes (DEGs) were randomly selected and analysed by RT-qPCR. We found that MSTRG.62661 and its putative target gene were co-expressed and significantly up-regulated under severe water stress (Additional file 9). Furthermore, MSTRG.18766 and its putative target gene were down-regulated under severe water stress. Meanwhile, the expression of MSTRG.22617.1 and target gene were down-regulated under water stress (Additional file 9). These results were relatively consistent with RNA-seq, indicating that lncRNA may participate in improving tolerance to water stress by regulating the expression of target gene.

**Table 1:** Statistic data of annotation lncRNAs and target genes

Annotation Type	Number of lncRNAs	Target genes	Expressed target genes	Differentially expressed target genes	DE-lncRNAs	Tagret genes of DE-lncRNAs	Differentially expressed target genes of DE-lncRNAs
Cis-target	3332	31,812	27,455	9321	449	6365	1921
Trans-target	161	202	166	75	20	24	5

**Fig. 4:**
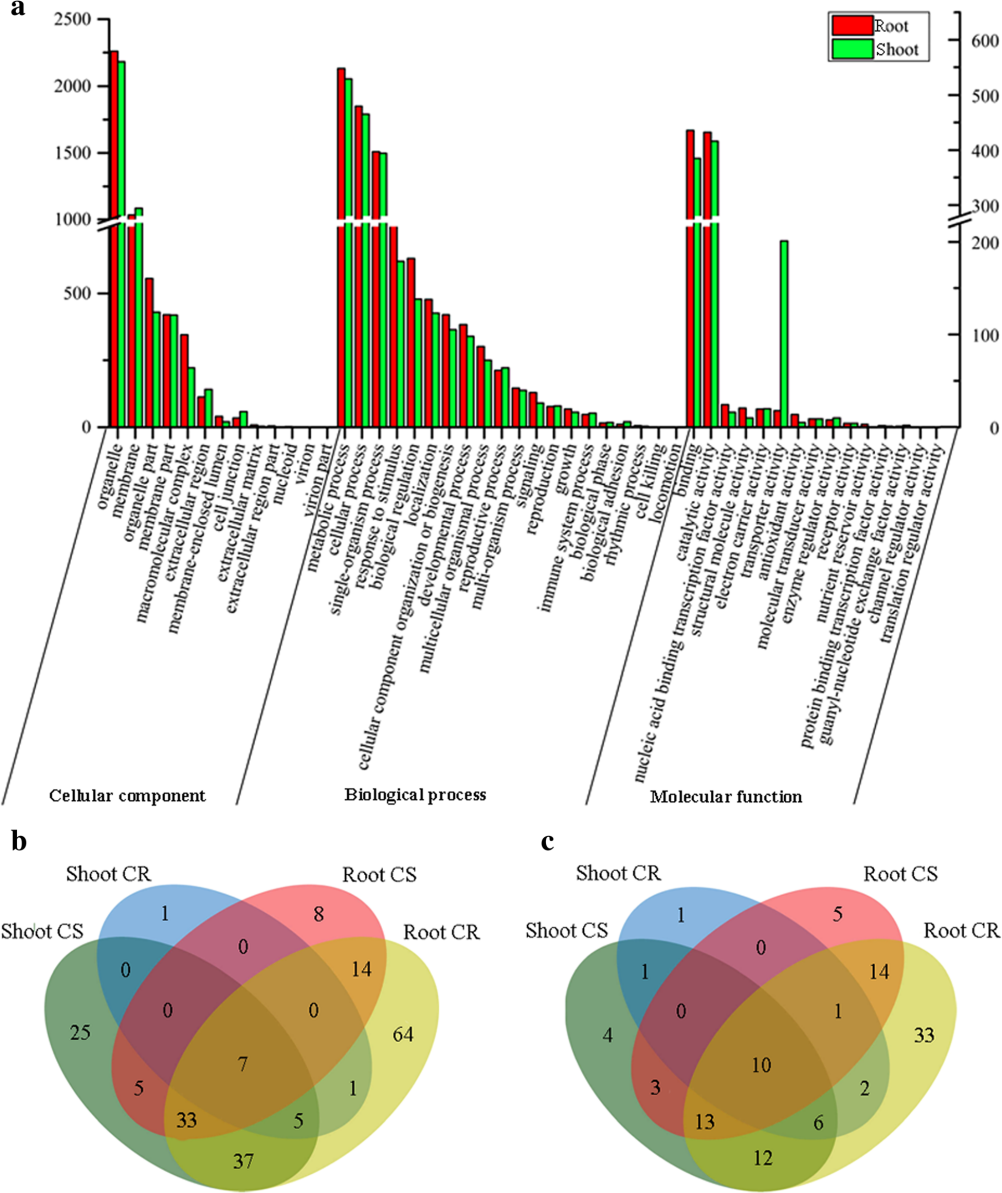
Functional analysis of DE-lncRNAs under water stress and during recovery. **a** Gene Ontology enrichment of co-expressed PCgenes with the DE-lncRNAs. Left data and right data are sorted by number of DE-lncRNAs target genes in root and shoot, respectively. *Red bar* DE-lncRNAs target genes in root; *green bar* DE-lncRNAs target genes in shoot. Venn diagram of significantly enriched GOs. The GO terms which were overrepresented under different conditions. **b** Biological process; **c** Molecular function. *CR*: control vs. recovery; *CS*: control vs. severe water stress

Compared to shoots, there was a large difference in number of DE-lncRNAs target genes in the roots (5616 target genes in the roots vs. 1503 target genes in the shoots; Additional file 10). The 731 common target genes were found in the two organs, including 81 DEGs. To decipher the major biological processes that are affected by water stress, gene ontology (GO) enrichment of the target genes and DEGs was performed on the two organs. GO analysis showed that the target genes and DEGs were mainly enriched for stress-related categories, such as response to stimulus, binging, biological regulation, catalytic activity, metabolic process and organelle (Fig. 4a and Additional file 10). Furthermore, the categories of biological adhesion and biological phase (for biological process) and nutrient reservoir activity (for molecular function) were enriched in target gene of DE-lncRNAs, while collagen trimer (for cellular components) was significantly in DEGs that is not associated with lncRNAs (Fig. 4a and Additional file 10). For cellular component analysis, the organelle and membrane were enriched in DE-lncRNAs target genes and DEGs that is not associated with lncRNAs, while nucleoid and collagen trimer was enriched only in DEGs that is not associated with lncRNAs. The response to stimulus and metabolic process were also enriched (Fig. 4a and Additional file 10).

To determine the target genes’ functional classes, which were chiefly involved in the response to water stress and recovery treatments, the significantly enriched GO terms were further selected by a mean *P*-value < 0.05. We also analysed the number of GO terms that were significantly enriched and in common between comparisons to determine differences and similarities between organs and conditions. Two comparisons were performed, CK vs. SS (CS) and CK vs. R (CR). The seven biological processes and ten molecular functions included among the significantly enriched GO terms were consistently over-represented for CS shoot, CS root, CR shoot, and CR root (Fig. 4b, c and Additional file 11). For example, GO terms were enriched for biological processes (GO:0006468, protein phosphorylation; GO:0006412, translation; and GO:0044242, cellular lipid catabolic process) and molecular functions (GO:0005524, ATP binding; GO:0005525, GTP binding; GO:0004674, protein serine/threonine kinase activity; and GO:0003676, nucleic acid binding).

Compared to specific biological functions for CS root, CS shoot GO term enrichment in response to water stress was dominated by functions related to the polyamine metabolic process (GO:0006595), photosynthesis (e.g., GO:0009768, photosynthesis, light harvesting in photosystem I), signal transduction (e.g., GO:0016024, CDP-diacylglycerol biosynthetic process and GO:0046341, CDP-diacylglycerol metabolic process), stomatal regulation (GO:0010440: stomatal lineage progression) and transport process (e.g., GO:0010496, intercellular transport; GO:0016482, cytoplasmic transport; and GO:0016192, vesicle-mediated transport; Fig. 4b). Among the specific molecular functions, specific GO terms for CS shoot included many enzyme activities (e.g., GO:0016682, oxidoreductase activity, acting on diphenols and related substances as donors; GO:0045485, oxygen as acceptor, omega-6 fatty acid desaturase activity; GO:0052745, inositol phosphate phosphatase activity; and GO:0008466, glycogenin glucosyltransferase activity); similarly, the specific GO terms for CS root also included many enzyme activities (e.g., GO:0004364, glutathione transferase activity, oxidoreductase activity; GO:0016706, acting on paired donors; and GO:0016773, phosphotransferase activity; Fig. 4c).

Compared to the specific molecular functions and biological functions for the CS shoot, shoot specific GO terms in CR only enriched for cysteine-type peptidase activity and alcohol metabolic process. The majority of GO terms were, however, specific to shoots and roots, and the transcriptional response in the CR root involved more functions than in the CS root. These GO terms included sugar transport (e.g., GO:0015770, sucrose transport; GO:0015786, UDP-glucose transport; and GO:0015758, glucose transport), tissue development (e.g., GO:0010067, procambium histogenesis and GO:0010065, primary meristem tissue development), signalling (GO:0019932, second-messenger-mediated signalling), nutrient reservoir (GO:0045735, nutrient reservoir activity), peroxisome (e.g., GO:0005053, peroxisome matrix targeting signal; GO:0000268, peroxisome targeting sequence binding; and GO:0004602, glutathione peroxidase activity), and oxidoreductase activity (e.g., GO:0052716, hydroquinone: oxygen oxidoreductase activity and GO:0016901, oxidoreductase activity; Fig. 4b and c). These results showed that the DE-lncRNAs regulated PCgenes and were involved in several important biological processes and molecular functions under water stress and during recovery.

### Metabolic pathways related to water stress and recovery in *C. songorica*

**Fig. 5:**
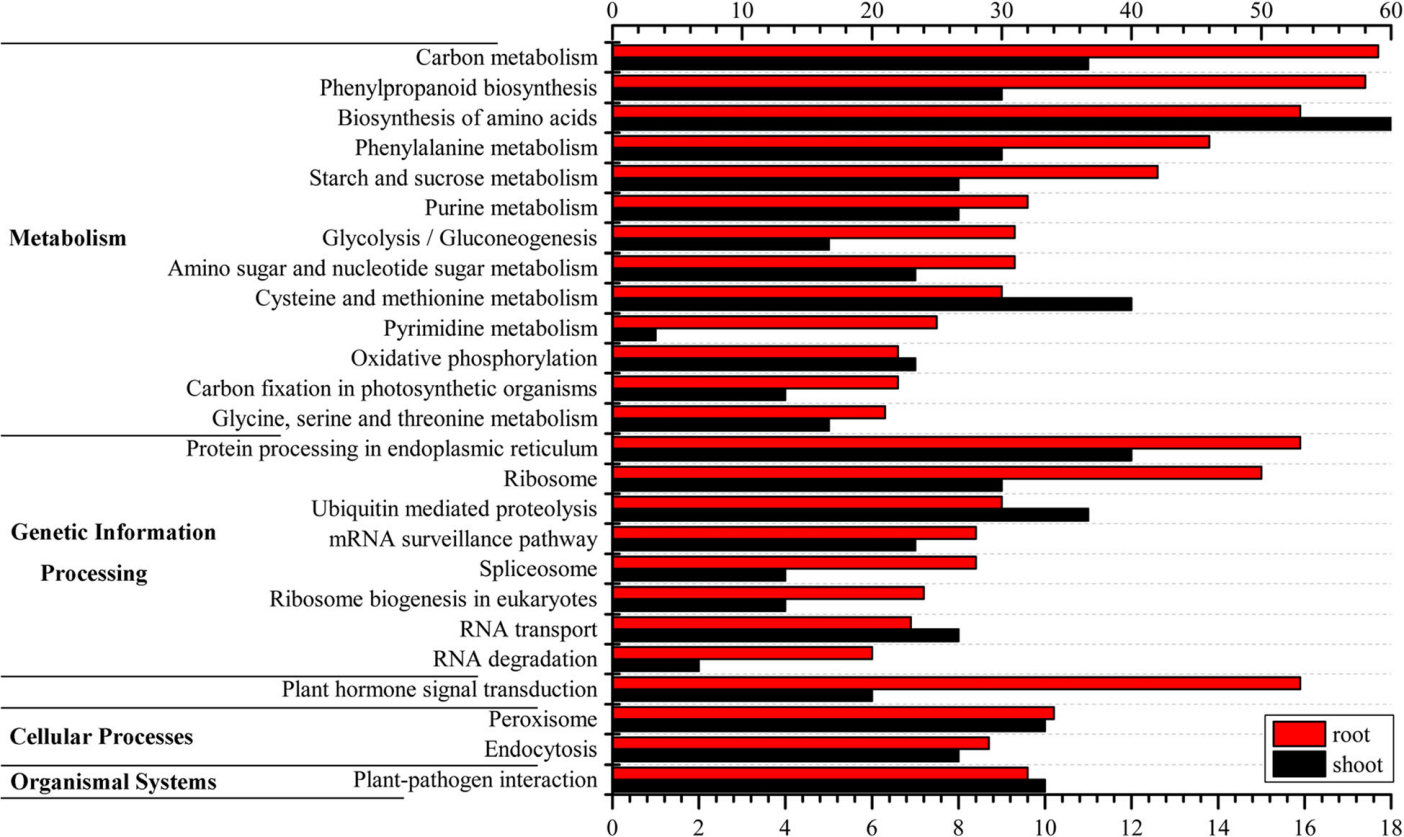
Distribution KEGG pathways for DE-lncRNAs target genes in root and shoot. Upper and lower data are sorted by number of DE-lncRNAs target genes in root and shoot mapping to KEGG pathways, respectively. Only categories with more than 20 DE-lncRNAs target genes in root are shown. *Red bar* root DE-lncRNAs target genes; *black bar* shoot DE-lncRNAs target genes

The set of 15,784 DEGs and 6388 DE-lncRNAs target genes were mapped with KEGG pathways in *C. songorica*, highlighting the involvement of several drought-related pathways (Fig. 5 and Additional file 9). Two important pathways focused on phenylpropanoids, including ‘phenylpropanoid biosynthesis’ and ‘phenylalanine metabolism’ were found to be regulated by water stress and during recovery in our study (Fig. 5; Additional file 12). However, the two terms had more DEGs in roots than in shoots. ‘Plant hormone signal transduction’, which comprised 141 DEGs (6 DE-lncRNAs target genes) and 153 DEGs (53 DE-lncRNAs target genes) in shoots and roots, respectively, was over-represented. Most of these genes belonged to the PYL and SnRK2 protein families. Strikingly, the biggest differences between the roots and shoots were related to ‘ribosomes’. The number of DEGs for ribosome were 3 times higher in shoots than in roots (Additional file 12). Reprogramming of ribosomal translation was identified as one of the largest responses of the shoot system under water stress; it can also be regulated by DE-lncRNAs in *C. songorica*. Additionally, DE-lncRNA target genes and DEGs were also involved in ‘starch and sucrose metabolism’, ‘ascorbate and aldarate metabolism’, ‘glutathione metabolism’, ‘arginine and proline metabolism’ and ‘fatty acid biosynthesis’ pathways, which are known to play important roles in plants resistance to abiotic stress.

### Identification of transcription factors in response to water stress and recovery in *C. songorica*

Transcription factors (TFs) have been identified to play an important role in improving plant resistance to abiotic stresses. Mining of the DEGs for putative TFs lead to the identification of 1644 DEGs, corresponding to 839 high-confidence rice homologues from 49 TF families (Additional file 13). A total of 523 of 839 rice homologues among the *C. songorica* DEGs (62%) were differentially expressed under drought stress in rice [36]. The DE-lncRNA target genes were also analysed to determine if any were included among the 1664 TFs. In total, 189 TFs corresponded to 163 *C. songorica* DE-lncRNAs (Additional file 13).

**Fig. 6:**
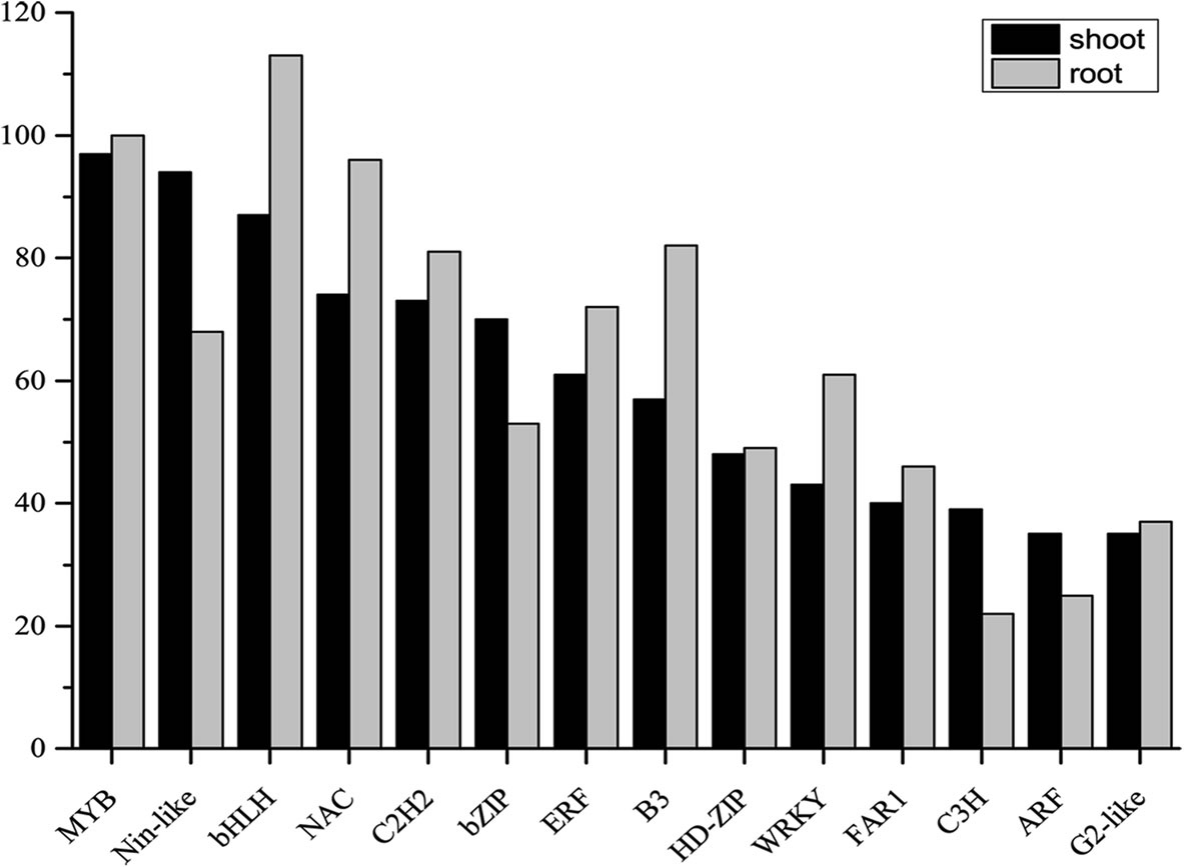
Distribution of transcription factors responsive to water stress and recovery in *C. songorica*. Data are sorted by number of DEGs in root. Only categories with more than 20 DEGs identified as transcription factors are shown. *Black bar* shoot DEGs; *gray bar* root DEGs

In *C. songorica*, MYB, bHLH, NAC, C2H2 and bZIP were most represented TFs families, which have been revealed to respond to drought stress in plants. The MYB family was the largest gene family among the identified families (144 in total), including 16 MYB genes as DE-lncRNA target genes (Fig. 6 and Additional file 13). The number of *C. songorica* MYB family in the roots and shoots was roughly the same. The BHLH, NAC, B3, and WRKY families showed a higher number of DEGs in the *C. songorica* roots compared to shoots. The BHLH family included 148 *C. songorica* DEGs corresponding to 82 rice homologous genes. Strikingly, 50 rice homologous to *C. songorica* DEGs were found to be differentially expressed also in rice. The NAC family genes from *C. songorica* were homologous to 60 rice NAC family genes, including *Os03g60080/SANC1*; *Os01g66120/SANC2/OsNAC6*; *Os11g03300/OsNAC10*; *Os08g06140*; *Os05g34830*; and *Os03g04070/ONAC022*. The 102 ERF, 17 AP2, and 5 RAV TF families constituted the AP2-EREBP superfamily that mediated abiotic and biotic stresses. The number of ARF, bZIP and C3H families in *C. songorica* were higher in the shoots than in the roots. Eleven of 90 bZIP genes were DE-lncRNA target genes, while five bZIP genes were homologous to rice *LOC_Os08g36790* and *LOC_Os06g10880* (Fig. 6 and Additional file 13).

### Characterization of co-regulated gene expression network in *C. songorica*

We compared the distribution of the differentially and non-differentially expressed *C. songorica* genes with the 15 drought-responsive modules recently identified as rice orthologues [37]. The putative orthologues among the *C. songorica* DEGs were further analysed as DE-lncRNAs target genes. Only Modules 3 and 4 were overrepresented in both shoots and roots (Table 2; Additional file 14). Module 1 was more represented in roots compared to shoots. Modules 2 and 5 displayed a higher number of differentially expressed putative orthologues in *C. songorica* shoots compared to roots. However, the functions of Modules 7, 9, 12, 13, 14 and 15 were not reported. Based on the GO enrichment results, Modules 15, 13, 12 and 10 might be related to ‘response to stimulus’, ‘fatty acid metabolic process’, ‘protein phosphorylation’ and ‘post-translational protein modification’, respectively (Table 2).

**Table 2:** Comparison between *C. songorica* water stress response genes and rice drought response network

Rice module	Rice genes	Puativa orthologs in *C. songorica*	Puativa orthologs in *C. songorica* target genes of DE_lncRNA	Puativa orthologs in *C. songorica* shoot DEGs	Puativa orthologs in *C. songorica* root DEGs	Puativa module function
Module 1	303	849	105	32/262	44/341	
Module 2	213	513	51	33/213	22/174	
Module 3	141	459	57	34/235	35/242	
Module 4	134	237	29	18/111	17/114	
Module 5	117	341	42	19/163	15/134	
Module 6	90	431	40	12/130	14/146	
Module 7	77	155	18	8/62	14/38	
Module 8	48	155	30	12/56	13/49	
Module 9	47	144	18	3/48	2/37	
Module 10	47	247	9	6/74	10/64	post-translational protein
Module 11	46	112	30	5/38	3/32	
Module 12	42	235	10	18/112	16/122	protein phosphorylation
Module 13	38	136	9	10/59	13/70	fatty acid metabolic process
Module 14	28	66	10	5/30	5/30	
Module 15	21	48	6	1/12	2/15	response to stimulus

There are 1392 rice genes in 15 modules. Base on OrthoMCL method, a total of 1605 and 1608 differentially expressed genes were identified as putative orthologues of rice genes in shoot and root of *C. songorica*, respectively. In putative orthologues in *C. songorica* shoot DEGs and root DEGs, the numbers on the left represent the DE_lncRNAs target genes in putative orthologs

### Identification of a core set of Poaceae genes that are differentially regulated upon water stress and during recovery

**Fig. 7:**
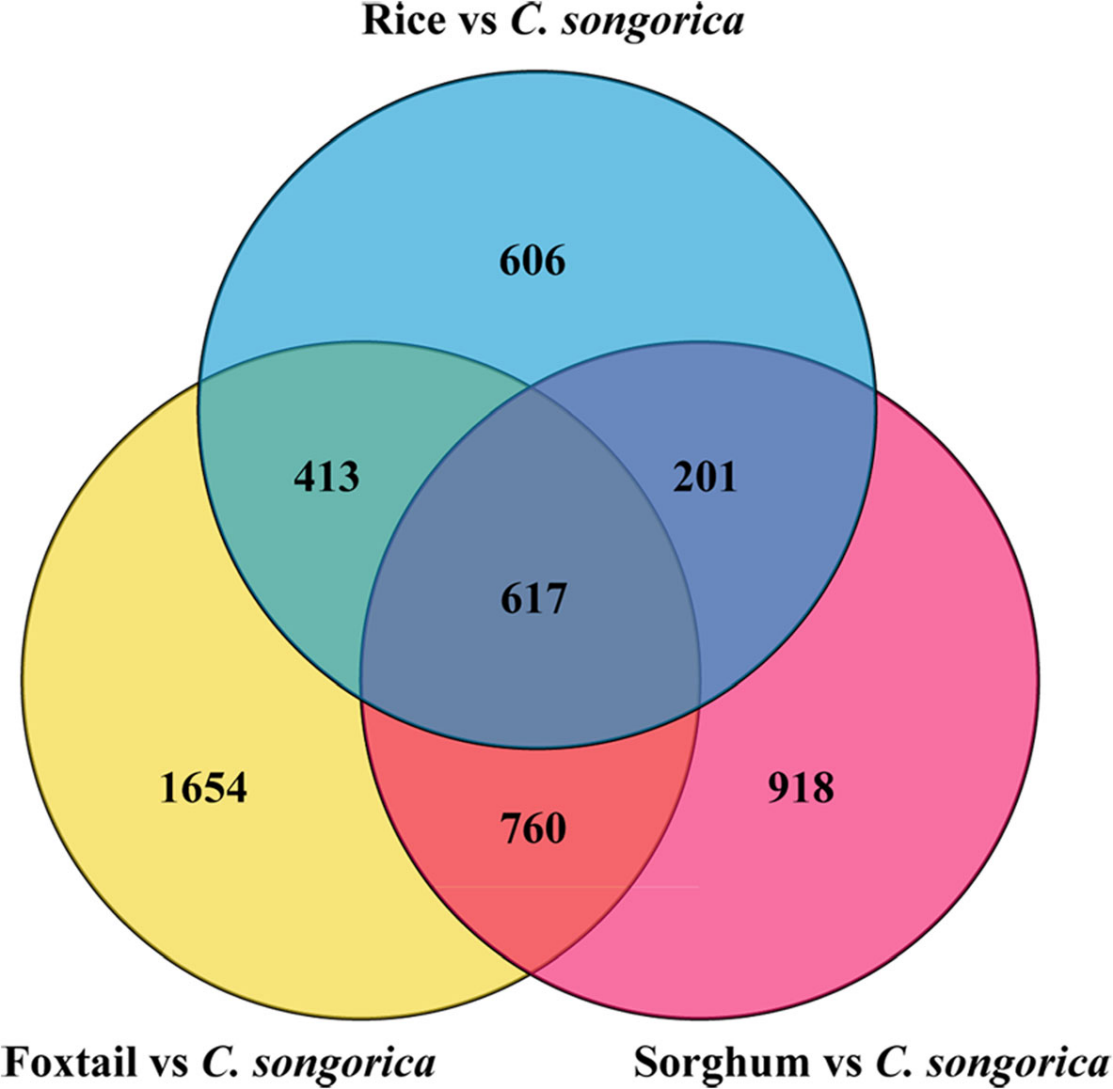
Drought response genes comparison across *C. songorica*, rice, foxtail, and sorghum. The Venn diagram represents putative orthologues of *C. songorica* stress-responsive genes identified by OrthoMCL in at least two species

The subset of water stress-related DEGs in common among *C. songorica*, foxtail, sorghum and rice were identified. A total of 2496, 3444, and 1837 putative orthologues were identified from foxtail, sorghum and rice, respectively (Fig. 7; Additional file 15). In total, 617 groups of putative orthologues present in all species were identified, which constitute a core of evolutionarily conserved genes associated with drought stress. Moreover, the conserved DEGs included 81 DE-lncRNAs target genes and 15 TFs (Additional file 15). These genes and lncRNAs will be used to establish the next network.

Several conserved genes participated in the biosynthesis of different metabolites, ranging from surge to lipids and flavonoids. The most interesting *C. songorica* candidates involved in ‘starch and sucrose metabolism’ were downregulated under water stress compared with the control condition. ‘Phenylpropanoid biosynthesis’ and ‘fatty acid elongation’ were the second functions of these conserved DEGs. Several other conserved DEGs participated in ‘ascorbate and aldarate metabolism’ and ‘arginine and proline metabolism’, which are known to be involved in responses to abiotic stress. Eight conserved DEGs involved in ‘proline metabolism’ were all differentially expressed under SS condition, but most of them were not differentially expressed under LS and R conditions. In agreement with previous studies, a certain number of membrane transporters were among the conserved DEGs involved in water stress. We found that 22 conserved DEGs were involved with ABC ‘transporter’. Another 18 conserved DEGs participated in ‘plant hormone signal transduction’. Among them contained proteins from the PP2Cs (protein phosphatase 2Cs) family that were enriched for ‘abscisic acid-activated signalling pathway’ and ‘protein phosphorylation’.

### *C. songorica* lncRNAs as endogenous target mimics for miRNAs

**Fig. 8:**
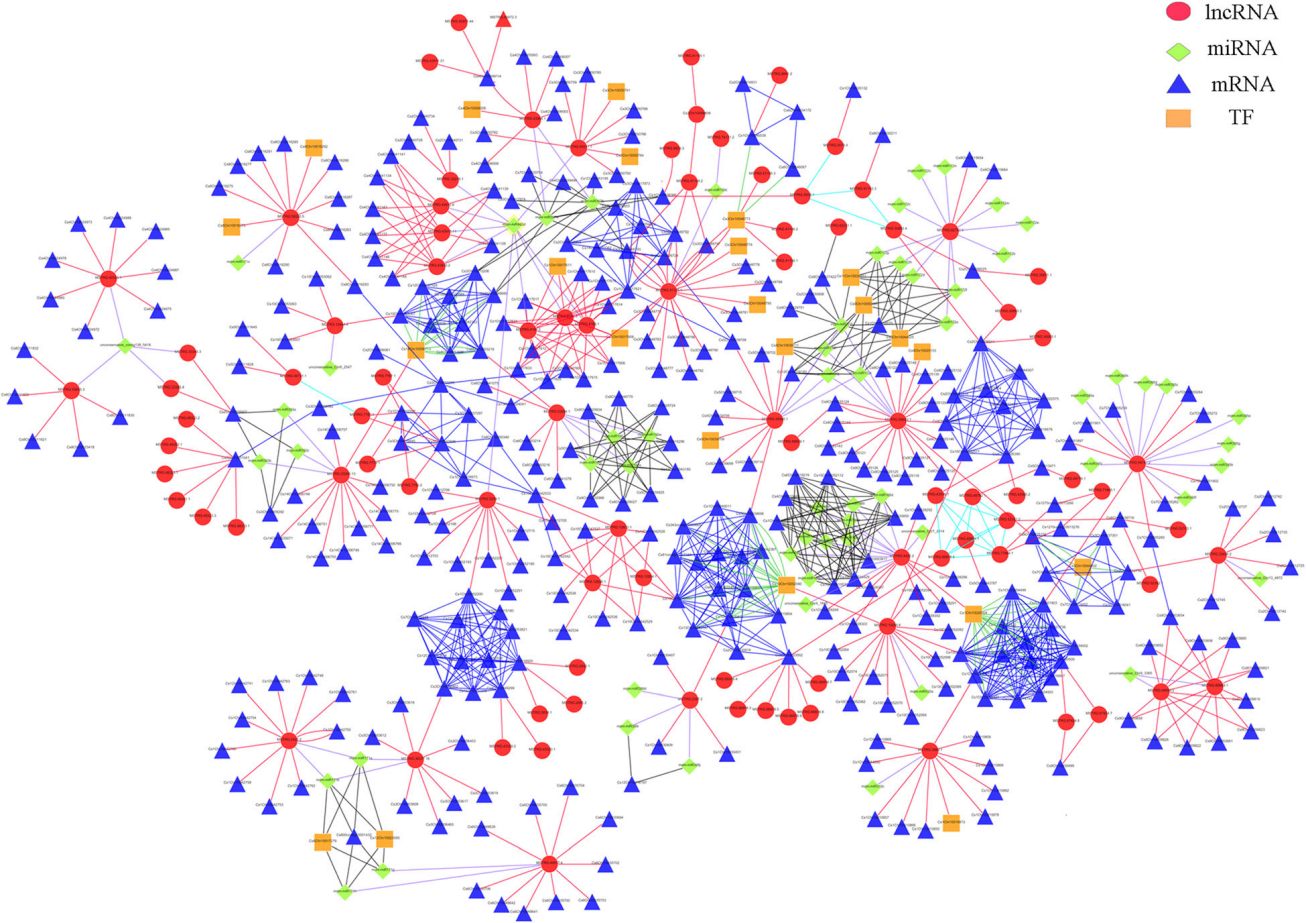
Representatives of predicted interaction networks among lncRNAs, PCgenes, miRNAs and transcription factors. The triangular, square, round and diamond nodes represent mRNAs, transcription factors, lncRNAs and miRNAs, respectively

**Fig. 9:**
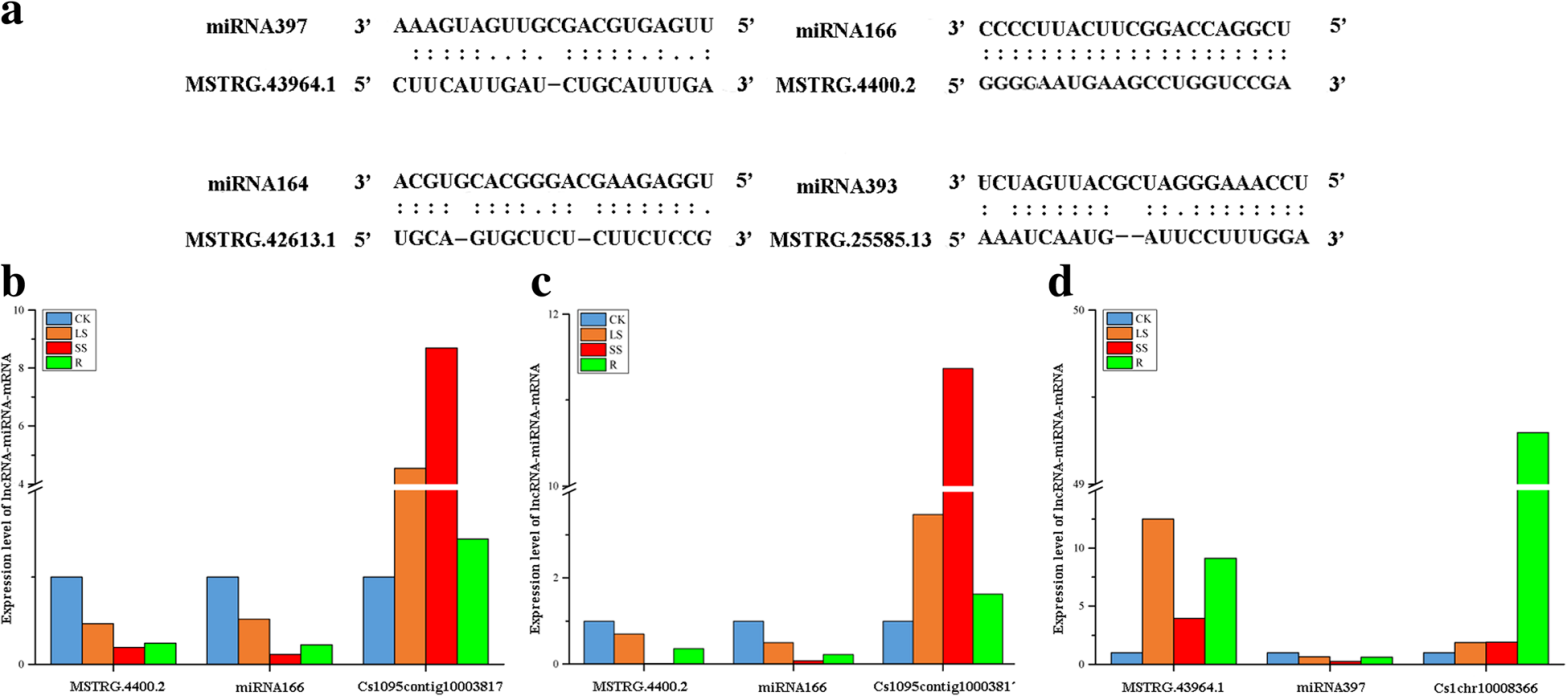
Functional prediction of *C. songorica* lncRNA as miRNA target mimics. **a** Predicted base-pairing interaction of miRNA-lncRNA. **b**-**d** Relative transcript abundances of lncRNA, miRNA and miRNA target gene in shoot (**b**) and root (**c**, **d**) under water stress and recovery. *CsGAPDH* and *U6* were used as the reference gene

In plants, an important function of lncRNAs is target mimicry; this miRNA-lncRNA relationship was discovered in *Arabidopsis*. In total, 52 of the identified lncRNAs may act as miRNA mimics by binding to known *C. songorica* miRNAs, including miRNA166, miRNA164, miRNA393, and miRNA397 (Additional file 16). We further constructed the co-expression network based on water stress-responsive lncRNAs, miRNAs, conserved DEGs and TFs from four Poaceae species. The result showed that lncRNAs, miRNAs, conserved DEGs and TFs constitute a complex transcriptional regulatory network based on some regulatory mechanism under water stress and recovery (Fig. 8). As shown in Fig. 8, miRNA397a and miRNA397b have seven target genes and bind to the MSTRG.43964.1 lncRNA (Figs. 8 and 9a). By sequence alignment, a drought-responsive lncRNA (MSTRG.4400.2) was found to bind to miRNA166 without mismatches and coordinated an increase in the expression of miRNA166 target genes under water stress (Figs. 8 and 9a). MSTRG.42613.1 also acted as a potential target mimic for conserved miRNA164, which regulated three NAC TFs (Fig. 8).

To further understand the relationship between the miRNA target mimics and the correlated miRNAs, lncRNA and corresponding miRNA expression were analysed under water stress and during recovery using RT-qPCR. We found the expression pattern of miRNA397 and MSTRG.43964.1 lncRNA were opposite (Fig. 9d). MSTRG.43964.1 and target gene of miRNA397 were up-regulated under water stress and recovery, but miRNA397 represented down-regulated. The evidences suggested MSTRG.43964.1 can up-regulate the target of miRNA397 expression by competing miRNA to down-regulated the activity of miRNA under water stress and recovery. Strikingly, the expression pattern of miRNA166 and MSTRG. 4400.2 lncRNA were identical in shoot and root (Fig. 9b and c). We suggested that fully complementary of miRNA166 and MSTRG.4400.2 has caused MSTRG. 4400.2 cleavage and up regulation of miRNA166 target gene.

## Discussion

With the advance in high-throughput sequencing tools, many novel lncRNA transcripts have been identified in different species [18, 38, 39]. These studies have revealed the complexity of eukaryotic gene expression and that lncRNA play important roles in many vital biological processes. Therefore, understanding the mechanisms of lncRNA regulation will provide a molecular basis for resistance research in plants. However, the genome identification and characterization of known and novel lncRNAs under drought stress in still lacking, especially in *C. songorica*. As a native plant, *C. songorica* has adapted to its harsh environment during its long evolutionary process. We recently completed *C. songorica* whole genome sequencing. MiRNAs have also been discovered in this plant [40]. In this study, stand-specific libraries were constructed to distinguish the sense and antisense lncRNAs. Moreover, the abundant clean data generated allowed us to detect low expression level lncRNAs in our research. To provide useful information for predicting putative lncRNA targets, PCgenes were also sequenced and compared in both shoots and roots under control and stress conditions.

In total, 3397 lncRNAs were identified in *C. songorica* in this study. The number of lncRNAs was more than was identified in maize and rice [17, 41], which may be due to the strict criteria or species differences. Compared to mRNAs, lncRNAs were shorter and had fewer exons in *C. songorica*. Moreover, *C. songorica* lncRNAs transcription levels were lower than that of mRNAs and lncRNAs were less conserved in different species. These results are consistent with previous results in other species [38, 42]. LncRNAs can modulate alternative splicing by hybridizing with target sense RNAs and blocking the recognition of the spliceosome splice site. Compared with 48.1% lncRNAs splicing in *Chlamydomonas reinhardtii* [43], 78.5% of *C. songorica* lncRNAs were spliced. Moreover, 412 and 87 lncRNAs were identified and differentially expressed in roots and shoots, respectively. However, the number of DEGs in different organs was roughly the same. This suggested that lncRNAs exhibit tissue-specific expression patterns.

Even though some lncRNAs have verified functions, the molecular mechanisms by which lncRNAs participate in bioprocesses are still largely unknown. For example, lncRNAs can regulate PCgenes transcription, post-transcription, and at post-translation levels [21]. Overall, several lncRNA mechanisms have emerged and have been classified as signalling, decoying, guiding and scaffolding [21]. Additionally, some lncRNAs have both *cis*- and *trans*-*acting* roles in regulating target genes. Because we had more than 5 samples, both *cis* and *trans* targeted genes were identified in this study. In total, 4885 and 722 specific target genes were identified in the roots and shoots, respectively. To understand the functions of the DE-lncRNAs under water stress and during recovery, we analysed the GO terms and KEGG pathway associated with the target genes, which were mainly enriched for stress-related categories. For example, ‘phenylpropanoid pathway’ and ‘plant hormones’ play crucial roles in the response to abiotic stress and were regulated by lncRNAs under water stress and during recovery in *C. songorica* [44, 45]. This indicated that lncRNAs play an important role in drought resistance mechanisms in *C. songorica*. To determine the similarity and differences between water stress and recovery in *C. songorica*, the significant enriched GO terms were compared, and 17 common GO terms were found between the different treatments and organs. There were more specific GO terms for water stress than during recovery in the shoots, but the opposite was true in the roots. This showed that there were differences in responses to water stress in the roots and shoots. For example, CS shoots were involved in photosynthesis, signal transduction, and transport process. In contrast, the CS root was enriched for tetrahydrofolate biosynthetic and metabolic, response to extracellular and external stimulus.

TFs are proteins that affect many biological processes including growth, development, cell division and response to environment stimuli, stressors in cells or organisms [46]. There was also a regulatory relationship between lncRNAs and TFs. For example, phytochrome-interacting factor 3 (PIF3) encodes a member of the (bHLH) TFs family. LncRNA HID1 (HIDDEN TREASURE 1) was shown to be an important player in seeding photomorphogenesis by modulating PIF3 expression [47]. In this study, 189 TFs corresponded to 163 DE-lncRNAs in *C. songorica*; for example, a bZIP gene was predicted to be a MSTRG.17203.1 target gene. Additionally, *C. songorica* genes homologues to rice genes, including *Os03g60080*(*SANC1*), *Os01g66120*(*SANC2*/*OsNAC6*), *Os11g03300*(*OsNAC10*), *Os08g06140*, *Os05g34830* and *Os03g04070*(*ONAC022*), were found to be involved in improving the drought stress tolerance of transgenic plants [48–53]. Five genes from the bZIP family were homologues of two rice genes (*LOC_Os08g36790* and *LOC_Os06g10880*), also called *OsbZIP66* and *OsbZIP46*, respectively, which had been demonstrated to improve drought tolerance in rice plants [54, 55]. In rice, these genes function within Module 10 and have been identified as involved in protein post-translational modification and the protein amino acid phosphorylation process [37]. In this study, these putative *C. songorica* DEGs orthologues were also mainly involved in phosphorylation in this module.

The expression patterns of orthologues in syntenic genomic blocks are likely to be correlated across species in the Poaceae family. Importantly, 617 pairs of orthologues were common between foxtail, sorghum, rice and *C. songorica* [37, 56, 57]. These orthologues may be a core group of evolutionarily conserved genes associated with drought stress. These conserved genes included 81 DE-lncRNAs target genes and 15 TFs. Strikingly, 5 conserved genes belonged to the homeodomain-leucine zipper (HD-Zip) TF family. Expression of the HD-Zip I genes were regulated by abiotic stress and hormones such as ABA and ethylene. *OsHOX22* (*LOC_Os04g45810*) and *OSHOX24* (*LOC_Os02g43330*), which belong to the HD-Zip I family, were found to be differentially expressed under abiotic stress conditions in rice [58]. Over-expressed *OSHOX24* enhanced the susceptibility to abiotic stress in transgenic rice [59]. Transgenic rice over-expressing *OSHOX22* showed increased sensitivity to ABA, increased ABA contents, and decreased drought and salt tolerance [60]. In this study, two conserved genes were homologous to *OSHOX24* and *OSHOX22*. Osmotic adjustment substances in plants such as soluble proteins, soluble sugars and proline were increased, which can improve plant drought resistance [61]. Our results showed that 8 conserved DEGs participated in proline metabolism. Strikingly, these DEGs were upregulated under water stress. Four conserved DEGs acted as DE-lncRNA target genes and were involved in starch and sucrose metabolism. The ABA signalling pathway was central to drought stress responses in plants. PP2Cs can be considered as ABA co-receptors [62]. Three DE-lncRNA target genes (two PP2C genes) were enriched for the plant hormone signal transduction pathway. ABA transportation required specific uptake transporters. Most ATP-binding cassette (ABC) proteins were integral membrane proteins and acted as ATP-driven transporters for a wide range of substrates, including lipids and auxin. For example, ABCG40 is a plasma membrane ABA uptake transporter in *Arabidopsis*. Stomata from *abcg40* mutants respond more slowly to ABA and the mutants showed decreased drought tolerance than wild plants [63]. Among these conserved genes, 18 conserved DEGs were involved in the ABA transporter pathway. Taken together, these results indicated that conserved genes play an important role in drought tolerance in *C. songorica*. ABA is the main plant hormone involved in responses to water stress. LncRNAs significantly participate in these important drought-resistant mechanisms in *C. songorica*.

The miRNAs have been clearly shown to act as post-transcriptional regulators of gene expression [64]. LncRNAs can act as miRNA precursors as well as interact with miRNAs as a competing endogenous RNA (ceRNA) or through target mimicry (TM) [65]. In *Arabidopsis*, miRNA399 is efficiently modulated by *IPS1*, which serves as endogenous sponge able to sequester miRNA399 [25]. Using bioinformatics, 52 lncRNAs were predicted to act as potential target mimics of conserved miRNAs in *C. songorica*. By analysing the expression of the lncRNA candidates and miRNA targets, one of these water stress-related lncRNAs (MSTRG.42613.1) was predicted to be a target mimic for miRNA164. It has been indicated that a decrease in miRNA164-target NAC genes causes drought tolerance in plant [66]. In this study, three miRNA164 NAC transcription factors were predicted as target genes. Auxin perception and signalling under normal growth conditions and during stress are regulated by miRNAs. TIR1 (transport inhibitor response1) is an auxin receptor that can be regulated by miRNA393. During stress, upregulated miRNA393 contributes to the repression of auxin signalling by promoting low TIR1 levels, leading to attenuation of plant growth and development under stress, and possibly promoting plant stress tolerance as well [67]. In this research, the lncRNA MSTRG.25585.13 was predicted to be a miRNA393 target mimic. Two miRNA393 target genes were annotated, including TIR1-like protein. There is significant ROS accumulation under abiotic stress conditions, which causes oxidative damage and eventually results in cell death [68]. However, recent work has indicated that ROS, especially H_2_O_2_, is an important second messenger in signal transduction networks that regulate plant development. Laccases (LACs) belong to a large group of enzymes termed blue copper proteins, which include ascorbic acid oxidase and plastocyanin. A previous study has shown that *OsLAC13* can produce H_2_O_2_ and is regulated by miRNA397 [69]. In this study, seven miRNA397a/b target genes were identified as being from the LAC family, including 2 laccase-13, 2 laccase-3, 2 laccase-10 and 1 laccase-4. MSTRG.43964.1 was predicted to be miRNA393a/b target mimic. Plants laccases are well-known to participate in lignin synthesis. In *Arabidopsis*, *LAC4* T-DNA insertion mutants have low lignin levels [70]. Taken together, our results suggest that *C. songorica* implements divergent mechanisms to modulate its response to water stress.

## Conclusion

In this study, 3397 lncRNAs were identified and 468 were differentially expressed under water stress and during recovery in *C. songorica*. The basic genomic features of these lncRNAs and PCgenes were characterized. The lncRNAs may regulate the PCgenes through *cis*- and *trans*-*acting* interactions. We analysed the GO enrichment and KEGG pathways of the DE-lncRNA target genes and found that the genes were mainly enriched for stress-related categories. We also discovered that specifically expressed lncRNAs under water stress and during recovery may act as endogenous target mimics for conserved miRNAs in *C. songorica*. In *C. songorica*, 52 lncRNAs were identified able to act as a target mimic for miRNAs. MSTRG.43964.1 and MSTRG.4400.2 may regulate the expression of miRNA397 and miRNA166 as a target mimic under water stress and during recovery, respectively. These findings extend the current view on lncRNAs as ubiquitous regulators in *C. songorica* under water stress and during recovery conditions.

## Methods

### Plant materials and water stress treatments

Seeds of *C. songorica* were obtained from the *C. songorica* production field of Lanzhou university (103°08′N, 38°62′E), Minqin County, Gansu Province, China. Bleach-sterilized seeds of *C. songorica* were germinated in a sand/vermiculite (1:1, *v*/v) mixture in a growth chamber that was controlled at 28/24 °C for day/night temperatures, with an irradiance of 150 μmol quanta m^− 2^ s^− 1^, 16 h light-8 h dark cycles and 65% relative humidity. Four-week-old seedling were transplanted into plastic basins, one plant per pot. Each pot was filled with the sand/vermiculite (1:1, v/v) mixture, with 0.45 kg per pot. Each plant was irrigated with 100 mL Hoagland nutrient solution every 3 days. Pot and artificial water control method were used to simulate the drought. After 28 days of growth, 36 pot seedings were randomly divided into the following six groups: control group (CK), light water stress group (LS), moderate water stress group (MS), severe water stress group (SS), recovery 4 h group (R4h) and recovery 48 h group (R48h). The initial soil moisture content of CK remained unchanged (soil water content is 95–100%), while stop watering the LS, MS and SS until the soil water content was decreased to 6–10%, 3–6% and 1–3%. The process took 2 weeks. The soil water content was tested every 2 days to replenish the amount of deficiency. For the R4h and R48h, the seedings were re-watered after the SS treatment for 4 h and 48 h. Shoots and roots from the control and treated groups were collected, immediately frozen in liquid nitrogen and stored at − 80 °C until used for RNA extraction. Six biological replicates were performed for each sample.

### Strand-specific RNA library construction and RNA sequencing

Total RNA from each independent sample was isolated using the TRIzol reagent (Invitrogen, USA) according to the manufacturer’s instructions. First, ribosomal RNA was removed with the Epicentre Ribo-zero™ rRNA Removal Kit (Epicentre, USA) and the rRNA-free residue was cleaned by ethanol precipitation. Subsequently, sequencing libraries were generated using the rRNA-depleted RNA with the NEBNext® Ultra™ Directional RNA Library Prep Kit for Illumina® (NEB, USA) following the manufacturer’s recommendations. Finally, the qualified cDNA libraries were constructed by PCR enrichment and sequenced on a HiSeq2500 with a sequencing read length of PE125. The library preparation and deep sequencing were performed by the Novogene Bioinformatics Technology Cooperation (Beijing, China). Clean reads were obtained by removing reads containing adapters, reads containing poly-N and lowquality reads from the raw reads. The clean reads were mapped to the *C. songorica* genome (data are not published) using HISAT2 (http://ccb.jhu.edu/software/hisat2/index.shtml) from the BMK Cloud server [71].

### LncRNA identification

The transcriptome was assembled using StringTie v1.3.1 (https://ccb.jhu.edu/software/stringtie/index.shtml) based on the clean reads mapped to the reference genome[72]. Following transcriptome assembly, the assembled transcripts were annotated using the gffcompare program. The following five steps were used to identify the lncRNA based on their characteristics [73, 74]: (1) select transcripts with a transcript class_code of “i”, “x”, “u”, “o”, or “e”; (2) transcripts with length ≤ 200 bp and an exon count ≤2 were removed; (3) transcripts with an FPKM ≥0.1 were selected; (4) transcripts encoding proteins and protein-coding domains were removed by alignment in the Pfam databases (http://pfam.xfam.org/); and (5) transcripts were eliminated that did not pass the protein-coding-score test using the Coding Potential Calculator (CPC, http://cpc.cbi.pku.edu.cn/), Coding-Non-Coding Index (CNCI, http://www.ncbi.nlm.nih.gov/pubmed/23892401) and the Coding Potential Assessment Tool (CPAT, http://lilab.research.bcm.edu/cpat/) [75–78]. The intersection of transcripts filtered by Pfam, CNCI, CPC and CPAT were considered as the resulting lncRNAs. The different types of lncRNAs, including lincRNAs, intronic lncRNAs, anti-sense lncRNAs, and sense lncRNAs, were selected using cuffcompare. To evaluate the sequence conservation, lncRNAs identified in *C. songorica* were used as the query data set in a BLASTN search against the genomes of other species, including, *Arabidopsis*, *O. sativa*, *Brachypodium distachyon* and *Medicago truncatula* (retrieved from Phytozome 12.0; https://phytozome.jgi.doe.gov/pz/portal.html#). The searches were performed with a cutoff query E ≤ 10^− 5^ and Qcov value ≥20. This analysis was performed using the BMK Cloud sever, an online platform for data analysis (http://www.biocloud.net/).

### Identification of DE-lncRNAs and DEGs

StringTie (1.3.1) was used to calculate FPKMs of both lncRNAs and mRNAs in each sample [79]. Differential expression analysis was performed using the DESeq R package (v1.10.1, negative binomial distribution). The resulting FDR (false discovery rate) were adjusted using the PPDE (posterior probability of being DE). The FDR < 0.01 & |log2(FoldChange)| ≥2 were set as the threshold for significantly differential expression.

### Analysis of DE-lncRNAs and DEGs function

To analysis the potential functions of DE-lncRNAs, we searched for protein coding genes spaced less than 100 kb away from the upstream and downstream of lncRNAs to predict putative lncRNAs target genes, and analyzing the complementary base-pairing between antisense lncRNAs and mRNAs using LncTar software [35].

GO enrichment analysis of PCgenes was implemented by the GO seq R package [80]. GO terms were identified to be significantly enriched with a *P*-value cutoff of 0.05. KOBAS software was used for testing the statistical enrichment of DE-lncRNAs target genes and DEGs in KEGG pathways [81].

### Identification of transcription factor

Transcription factor analysis was performed using the BMK Cloud sever, an online platform for data analysis (http://www.biocloud.net/).

### Identification of conserved genes in response to water stress in Poaceae

To identify the subsets of water stress-related DEGs in common among *C. songorica*, foxtail, sorghum and rice, OrthoMCL software V5 was used with default settings [82].

### Prediction of lncRNAs as miRNA target mimics

Targets were predicted by submitting all the known *C. songorica* miRNA and all lncRNAs to psRNATarget (http://plantgrn.noble.org/psRNATarget), with less than three mismatches and G/U pairs allowed within the lncRNA and miRNA pairing regions [83]. Mature *C. songorica* miRNAs sequences came from our previous studies [40]. The co-expression network was established using Cytoscape (http://cytoscapeweb.cytoscape.org/), based on the conserved genes, target genes of miRNAs, DE-lncRNAs and target genes of DE-lncRNAs.

### Quantitative real-time (RT) PCR

Total RNA was isolated respectively from *C. songorica* shoot and root after stress treatments for qRT-PCR using RNAiso regent (TaKaRa, Dalian, China). The qRT-PCR was performed using SYBR Premix Ex TaqTM (TaKaRa). About 1 μg RNA was reverse-transcribed into first-stand cDNA with PrimeScript® RT reagent Kit (TaKaRa), and product was used as templated for RT-qPCR using specific primers (Additional file 17). The *CsGAPDH* and *U6* were used to the reference gene. The relative expression levels were calculated by the comparative CT method. All reaction was performed in triplicate.

## Additional files

Additional file 1: Physiological analysis of *C. songorica* in response to water stress and recovery. (DOCX 210 kb)

Additional file 2: RNA-seq data for eighteen samples. (XLSX 10 kb)

Additional file 3: The box plot of expression levels of lncRNAs and mRNAs under different conditions in root and shoot, respectively. (DOCX 930 kb)

Additional file 4: Numbers of lncRNAs conserved in *C. songorica*, Arabidopsis, *Brachypodium distachyon* and *Medicago truncatula* and rice (*Oryza sativa*). (DOCX 1598 kb)

Additional file 5: The abundance of specifically expressed genes and specifically expressed lncRNAs (FPKM). (DOCX 360 kb)

Additional file 6: Significantly DEGs (FDR ≤ 0.01) in at least one stress treatment and their regulated in shoot and root. (XLSX 2110 kb)

Additional file 7: Significantly DE-lncRNAs (FDR ≤ 0.01) in at least one stress treatment and their regulated and target genes in shoot and root. (XLSX 1061 kb)

Additional file 8: List of all LncRNAs and target genes. (XLSX 1554 kb)

Additional file 9: The analysis of RT-qPCR with lncRNA and their putative target genes (DEG). (DOCX 338 kb)

Additional file 10: Functional analysis of DEGs under water stress and recovery. (DOCX 755 kb)

Additional file 11: List of over-represented significantly enriched GO terms in common between different combinations of organ/condition comparisons corresponding to the Venn chart in Fig. 4b, c. (XLSX 29 kb)

Additional file 12: Distribution KEGG Pathways for DEGs in root and shoot. (DOCX 497 kb)

Additional file 13: Summary of differentially expressed TFs identified in *C. songorica* water-stressed transcriptome. (XLSX 205 kb)

Additional file 14: List of putative orthologues or rice genes in shoot and root of *C. songorica*. (XLSX 113 kb)

Additional file 15: List of common conserved drought responsive genes shared across 4 Poaceae species and their expression pattern in *C. songorica*. (XLSX 291 kb)

Additional file 16: Putative targets and target mimics of lncRNAs for miRNA in *C. songorica*. (XLSX 29 kb)

Additional file 17: Primer list for gene specific primers. (XLSX 11 kb)

## Abbreviations

ceRNAs: competing endogenous RNAs
CNCI: Coding-Non-Coding Index
CPAT: Coding Potential Assessment Tool
CPC: Coding Potential Calculator
eTMs: endogenous target mimics
FPKM: Fragments per kilobase of exon per million fragments mapped
LncRNAs: Long non-coding RNAs
miRNA: microRNA
PCgenes: Protein-coding genes
RT-qPCR: Real-time quantitative PCR
