# Supplementary material for: Differential co-expression networks of long non-coding RNAs and mRNAs in Cleistogenes songorica under water stress and during recovery
Source: BMC Plant Biol. 2019 Jan 11;19:23. doi: 10.1186/s12870-018-1626-5 (PMC6330494; doi:10.1186/s12870-018-1626-5)
Supplement: Supplementary file 1 — Physiological analysis of C. songorica in response to water stress and recovery. (DOCX 210 kb) [file 12870_2018_1626_MOESM1_ESM.docx]

**Physiological analysis of *C. songorica* in response to water stress and during recovery.**


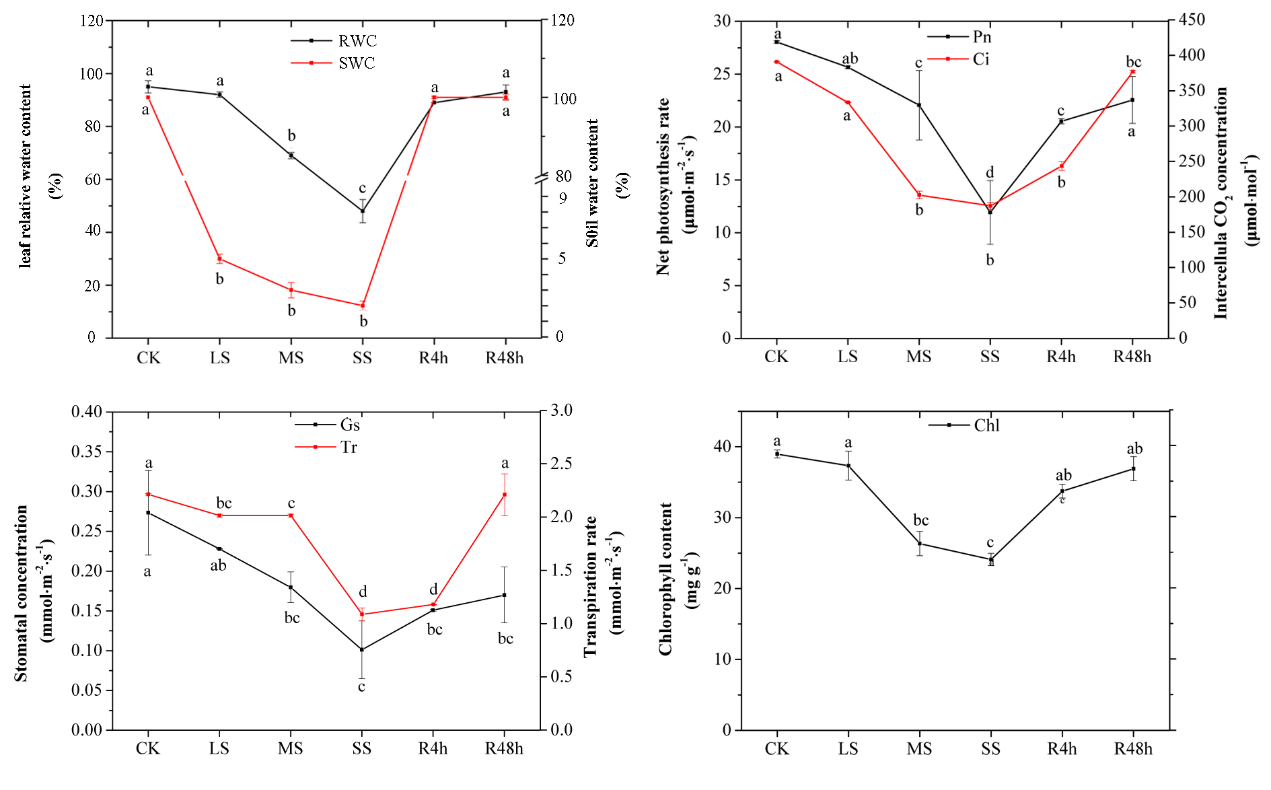


CK (control), LS (light drought stress), MS (Moderate drought stress), SS (severe drought stress), R4h (recovery 4h) and R48h (recovery 48h). Leaf relative water content (RWC), Photosynthesis rate (Pn), intercellular CO2 concentration (Ci), stomatal conductance (Gs), transpiration rate (Tr) and chlorophyll content (Chl) were examined in *C. songorica* under normal and treated conditions. Data are mean ± SD calculated from four independent experiments. In the same index, different lowercase letters indicate significant differences at 0.05 level.
