# Supplementary material for: Differential co-expression networks of long non-coding RNAs and mRNAs in Cleistogenes songorica under water stress and during recovery
Source: BMC Plant Biol. 2019 Jan 11;19:23. doi: 10.1186/s12870-018-1626-5 (PMC6330494; doi:10.1186/s12870-018-1626-5)
Supplement: Supplementary file 3 — The box plot of expression levels of lncRNAs and mRNAs under different conditions in root and shoot, respectively. (DOCX 930 kb) [file 12870_2018_1626_MOESM3_ESM.docx]

**The box plot of expression levels of lncRNAs and mRNAs under different conditions in root and shoot, respectively.**


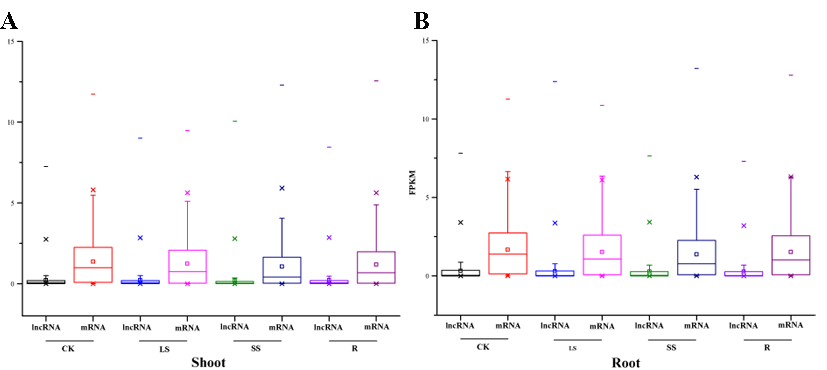


CK (control), LS (light drought stress), MS (Moderate drought stress), SS (severe drought stress) and R (recovery 48h).
