# Supplementary material for: Differential co-expression networks of long non-coding RNAs and mRNAs in Cleistogenes songorica under water stress and during recovery
Source: BMC Plant Biol. 2019 Jan 11;19:23. doi: 10.1186/s12870-018-1626-5 (PMC6330494; doi:10.1186/s12870-018-1626-5)
Supplement: Supplementary file 4 — Numbers of lncRNAs conserved in C. songorica, Arabidopsis, Brachypodium distachyon and Medicago truncatula and rice (Oryza sativa). (DOCX 1598 kb) [file 12870_2018_1626_MOESM4_ESM.docx]

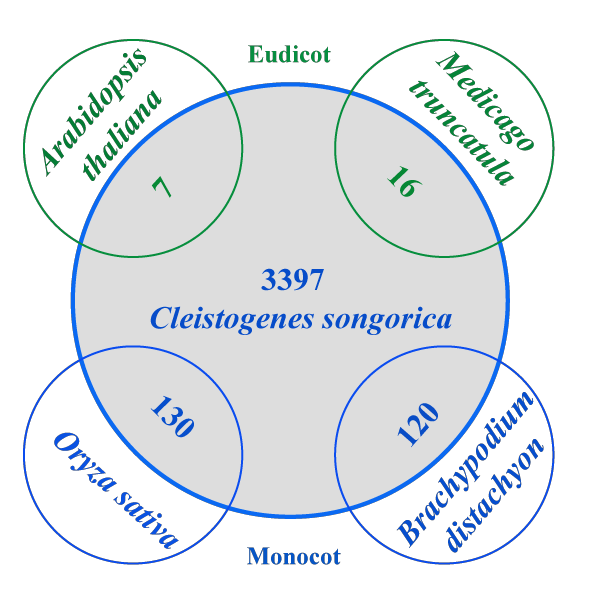


**Numbers of lncRNAs conserved in *C. songorica*, Arabidopsis, *Brachypodium distachyon* and *Medicago truncatula* and *Oryza sativa*.**
