# Supplementary material for: Differential co-expression networks of long non-coding RNAs and mRNAs in Cleistogenes songorica under water stress and during recovery
Source: BMC Plant Biol. 2019 Jan 11;19:23. doi: 10.1186/s12870-018-1626-5 (PMC6330494; doi:10.1186/s12870-018-1626-5)
Supplement: Supplementary file 5 — The abundance of specifically expressed genes and specifically expressed lncRNAs (FPKM). (DOCX 360 kb) [file 12870_2018_1626_MOESM5_ESM.docx]

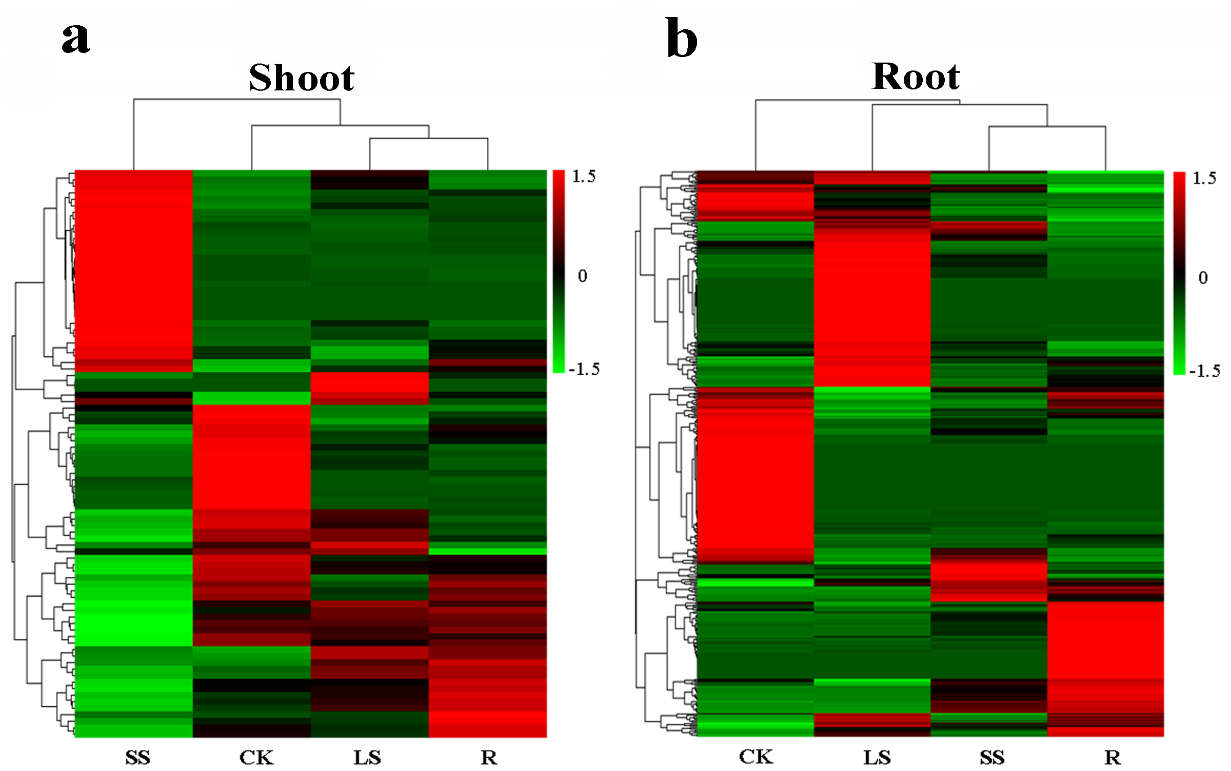


**The abundance of specifically expressed lncRNAs (FPKM)**

The rows and columns were ordered according to Cluster3.0. (**a**) 87 specifically expressed lncRNAs in shoot. (**b**), 412 specifically expressed lncRNAs in root.


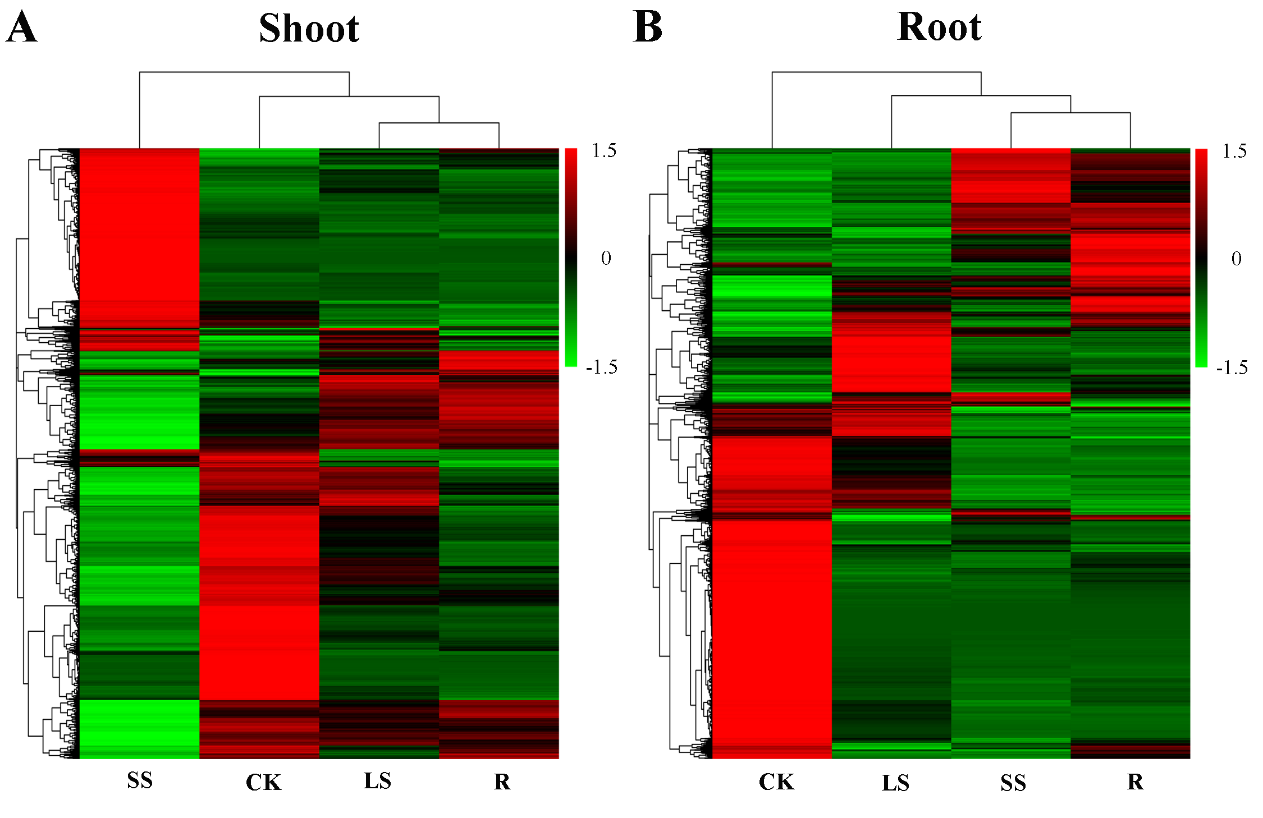


**The abundance of differentially expressed genes (FPKM).**

CK (Control), LS (Light drought stress), MS (Moderate drought stress), SS (Severe drought stress), and R (Recovery 48h)
