# Supplementary figures and images for: Differential co-expression networks of long non-coding RNAs and mRNAs in Cleistogenes songorica under water stress and during recovery
Source: BMC Plant Biol. 2019 Jan 11;19:23. doi: 10.1186/s12870-018-1626-5 (PMC6330494; doi:10.1186/s12870-018-1626-5)

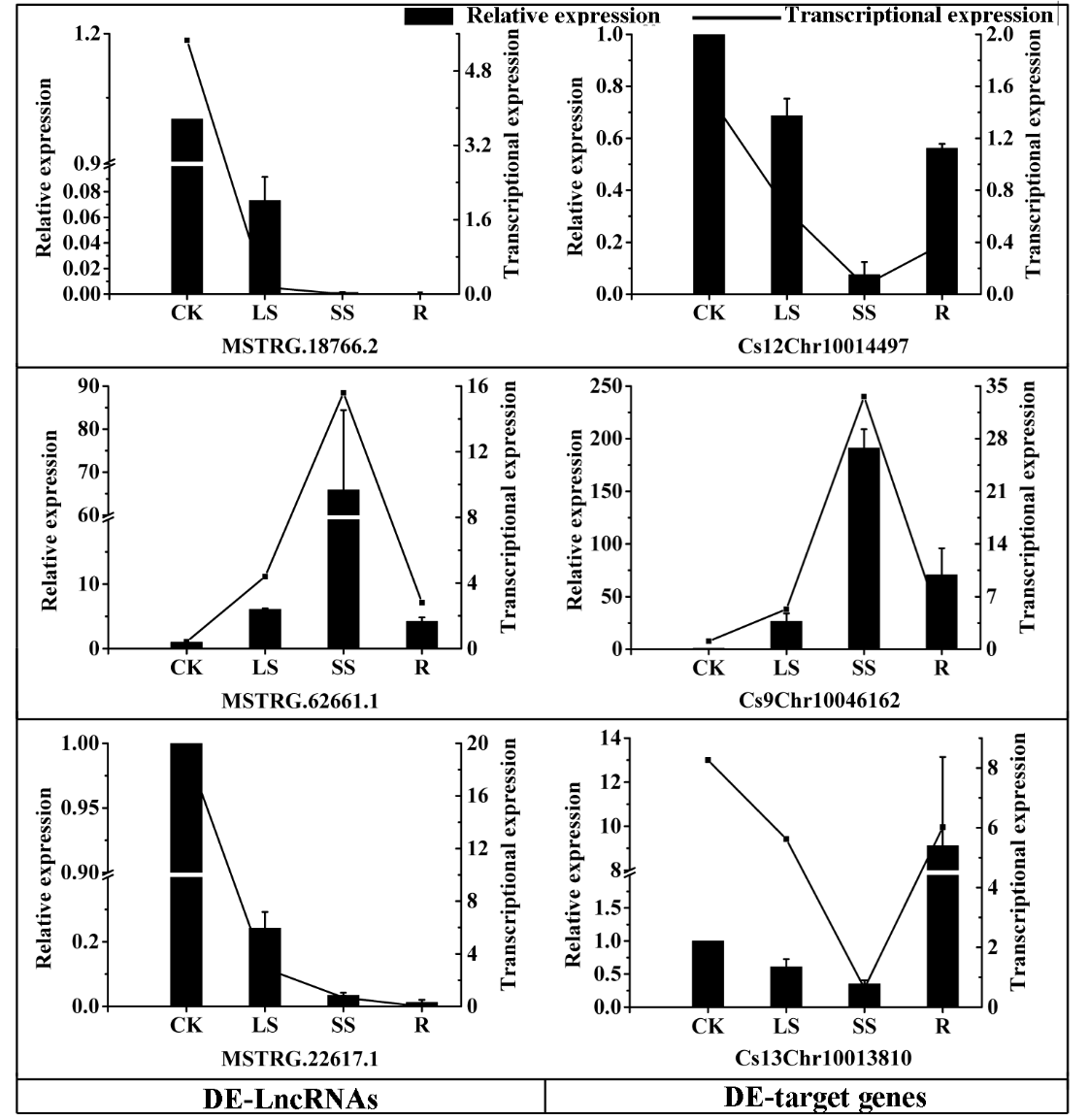


**The analysis of RT-qPCR with DE-lncRNA and their putative target genes (DEG).**

Supplement: Supplementary file 9 — The analysis of RT-qPCR with lncRNA and their putative target genes (DEG). (DOCX 338 kb) [file 12870_2018_1626_MOESM9_ESM.docx]

**Distribution KEGG Pathways for DEGs in root and shoot.**

**
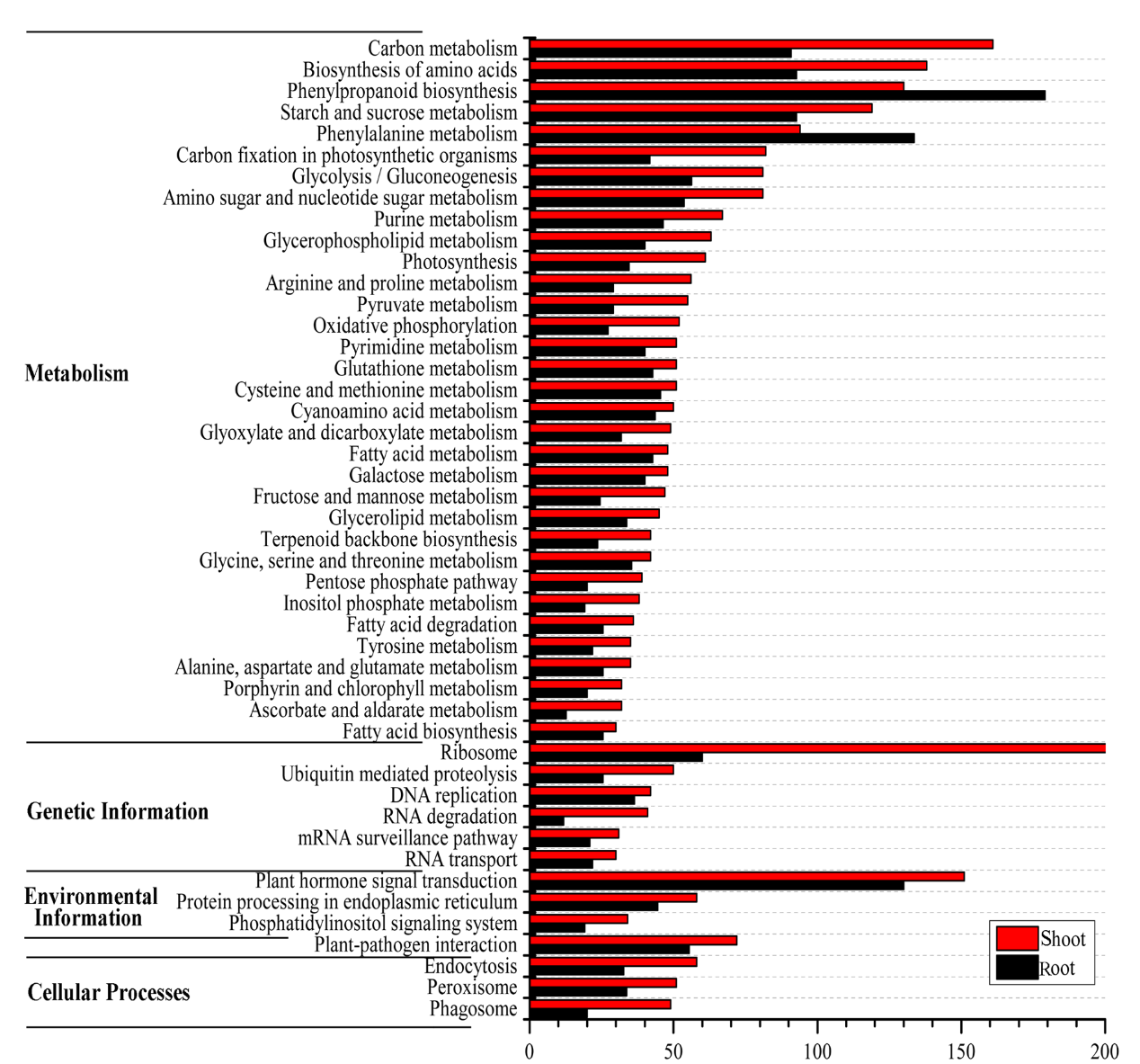
**

Supplement: Supplementary file 12 — Distribution KEGG Pathways for DEGs in root and shoot. (DOCX 497 kb) [file 12870_2018_1626_MOESM12_ESM.docx]
