# Supplementary material for: Differential co-expression networks of long non-coding RNAs and mRNAs in Cleistogenes songorica under water stress and during recovery
Source: BMC Plant Biol. 2019 Jan 11;19:23. doi: 10.1186/s12870-018-1626-5 (PMC6330494; doi:10.1186/s12870-018-1626-5)

**Functional analysis of differentially expressed genes under water stress and during recovery**


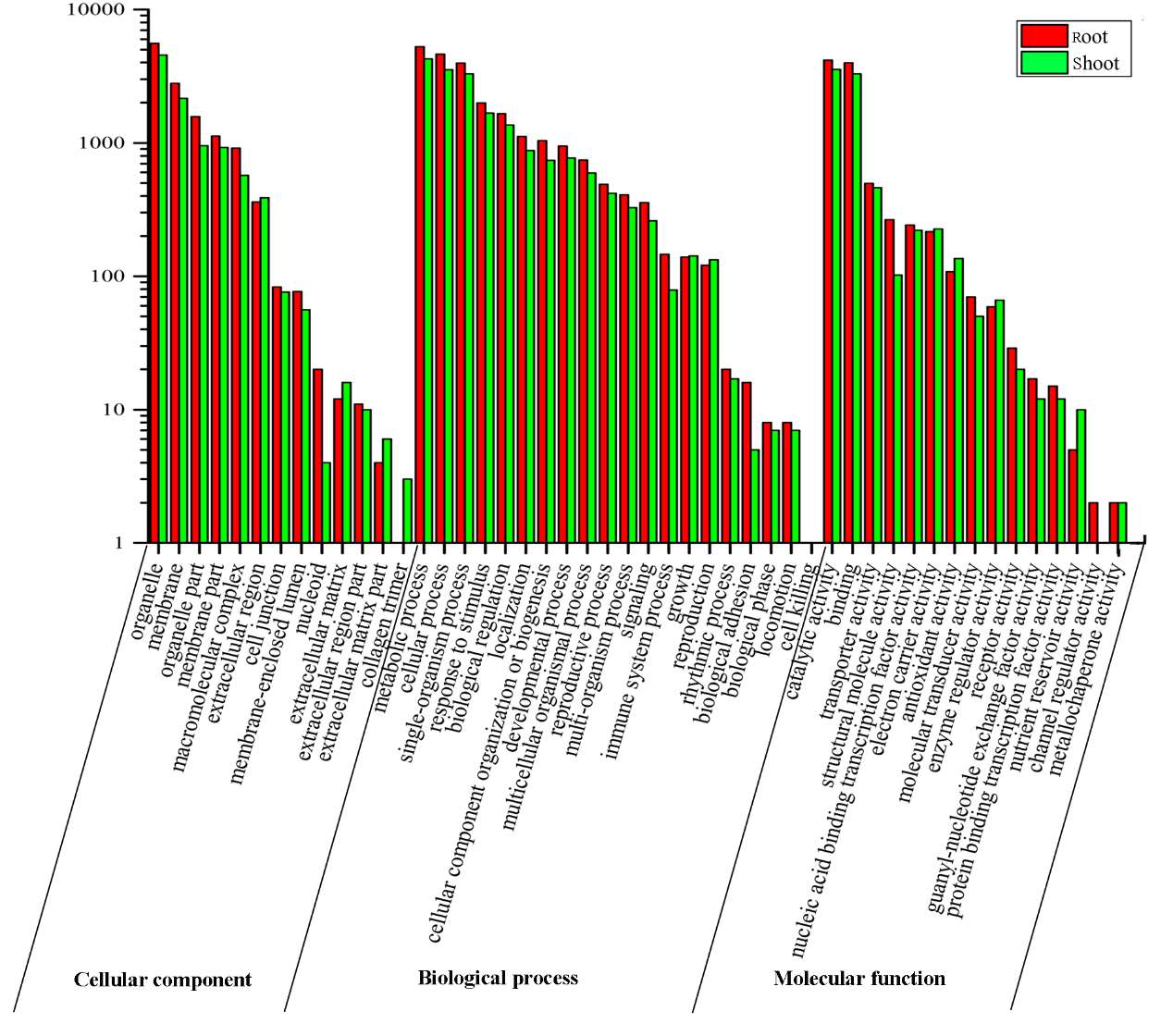

Supplement: Supplementary file 10 — Functional analysis of DEGs under water stress and recovery. (DOCX 755 kb) [file 12870_2018_1626_MOESM10_ESM.docx]
